# Leveraging pleiotropy to discover and interpret GWAS results for sleep-associated traits

**DOI:** 10.1371/journal.pgen.1010557

**Published:** 2022-12-27

**Authors:** Sung Chun, Sebastian Akle, Athanasios Teodosiadis, Brian E. Cade, Heming Wang, Tamar Sofer, Daniel S. Evans, Katie L. Stone, Sina A. Gharib, Sutapa Mukherjee, Lyle J. Palmer, David Hillman, Jerome I. Rotter, Craig L. Hanis, John A. Stamatoyannopoulos, Susan Redline, Chris Cotsapas, Shamil R. Sunyaev

**Affiliations:** 1 Division of Genetics, Brigham and Women’s Hospital, Boston, Massachusetts, United States of America; 2 Division of Pulmonary Medicine, Boston Children’s Hospital, Boston, Massachusetts, United States of America; 3 Altius Institute for Biomedical Sciences, Seattle, Washington, United States of America; 4 Department of Pediatrics, Harvard Medical School, Boston, Massachusetts, United States of America; 5 Department of Organismic and Evolutionary Biology, Harvard University, Cambridge, Massachusetts, United States of America; 6 Division of Sleep and Circadian Disorders, Brigham and Women’s Hospital, Boston, Massachusetts, United States of America; 7 Division of Sleep Medicine, Harvard Medical School, Boston, Massachusetts, United States of America; 8 Broad Institute of Harvard and MIT, Cambridge, Massachusetts, United States of America; 9 Department of Medicine, Harvard Medical School, Boston, Massachusetts, United States of America; 10 California Pacific Medical Center Research Institute, San Francisco, California, United States of America; 11 Division of Pulmonary, Critical Care, and Sleep Medicine, University of Washington, Seattle, Washington, United States of America; 12 Computational Medicine Core at Center for Lung Biology, University of Washington, Seattle, Washington, United States of America; 13 Respiratory and Sleep Services, Southern Adelaide Local Health Network, Adelaide, South Australia, Australia; 14 Adelaide Institute for Sleep Health, Flinders University, Adelaide, South Australia, Australia; 15 School of Public Health, University of Adelaide, Adelaide, South Australia, Australia; 16 Centre for Sleep Science, University of Western Australia, Perth, Australia; 17 Department of Pulmonary Physiology and Sleep Medicine, Sir Charles Gairdner Hospital, Perth, Australia; 18 The Institute for Translational Genomics and Population Sciences, Department of Pediatrics, The Lundquist Institute for Biomedical Innovation at Harbor-UCLA Medical Center, Torrance, California, United States of America; 19 Department of Epidemiology, Human Genetics and Environmental Sciences, School of Public Health, University of Texas Health Science Center at Houston, Houston, Texas, United States of America; 20 Departments of Medicine and Genome Sciences, University of Washington, Seattle, Washington, United States of America; 21 Division of Pulmonary, Critical Care, and Sleep Medicine, Beth Israel Deaconess Medical Center, Boston, Massachusetts, United States of America; 22 Department of Neurology, Yale School of Medicine, New Haven, Connecticut, United States of America; 23 Department of Genetics, Yale School of Medicine, New Haven, Connecticut, United States of America; 24 Department of Biomedical Informatics, Harvard Medical School, Boston, Massachusetts, United States of America; University of Oxford, UNITED KINGDOM

## Abstract

Genetic association studies of many heritable traits resulting from physiological testing often have modest sample sizes due to the cost and burden of the required phenotyping. This reduces statistical power and limits discovery of multiple genetic associations. We present a strategy to leverage pleiotropy between traits to both discover new loci and to provide mechanistic hypotheses of the underlying pathophysiology. Specifically, we combine a colocalization test with a locus-level test of pleiotropy. In simulations, we show that this approach is highly selective for identifying true pleiotropy driven by the same causative variant, thereby improves the chance to replicate the associations in underpowered validation cohorts and leads to higher interpretability. Here, as an exemplar, we use Obstructive Sleep Apnea (OSA), a common disorder diagnosed using overnight multi-channel physiological testing. We leverage pleiotropy with relevant cellular and cardio-metabolic phenotypes and gene expression traits to map new risk loci in an underpowered OSA GWAS. We identify several pleiotropic loci harboring suggestive associations to OSA and genome-wide significant associations to other traits, and show that their OSA association replicates in independent cohorts of diverse ancestries. By investigating pleiotropic loci, our strategy allows proposing new hypotheses about OSA pathobiology across many physiological layers. For example, we identify and replicate the pleiotropy across the plateletcrit, OSA and an eQTL of DNA primase subunit 1 (*PRIM1*) in immune cells. We find suggestive links between OSA, a measure of lung function (FEV_1_/FVC), and an eQTL of matrix metallopeptidase 15 (*MMP15*) in lung tissue. We also link a previously known genome-wide significant peak for OSA in the hexokinase 1 (*HK1)* locus to hematocrit and other red blood cell related traits. Thus, the analysis of pleiotropic associations has the potential to assemble diverse phenotypes into a chain of mechanistic hypotheses that provide insight into the pathogenesis of complex human diseases.

## Author summary

Large genetic studies with hundreds of thousands of patients have been successful at finding genetic variants that associate with disease traits in humans. However, smaller-scale studies can often have inadequate power to discover new genetic associations. Here, we use a small genetic study of Obstructive Sleep Apnea (OSA), to introduce a strategy that both helps find genetic associations and proposes biological hypotheses for the mechanisms behind those associations. To achieve this, we use large genetic studies carried out on traits that are related to OSA, and look for genetic variants that affect both OSA in our small study and the trait in question in the large study. By linking two or more traits at select loci, we were able to, among other results, find a locus that affects the expression of a gene in immune cells (DNA primase subunit 1), a marker of thrombotic and inflammatory processes (plateletcrit) and OSA. This results in a novel genetic association to OSA and a corresponding biological hypothesis behind its effect on OSA.

## Introduction

Genome-wide association studies of human phenotypes ranging from gene expression to human diseases are now routine. Cumulatively, the data indicate that complex traits are highly polygenic [1,2], and genetic correlation between these traits indicates abundant pleiotropy [3–5]. Interpreting the plethora of results raises two major challenges: first, generating testable mechanistic hypotheses about the underlying pathophysiology; and second, increasing statistical power to identify associations in studies of traits with small or moderate sample sizes. Leveraging pleiotropy can help address both of these challenges. Previous work has demonstrated that including many correlated traits in association studies increases power to detect associations common to multiple traits [3,6,7]. This approach is largely untried in genetic investigations of Obstructive Sleep Apnea (OSA). Here, we demonstrate that using shared associations between correlated traits can identify effects in under-powered studies of OSA, and that leveraging molecular and physiological endophenotypes in this way also generates clear and testable biological hypotheses.

OSA is characterized by recurrent episodes of partial or complete obstruction of the pharyngeal airway resulting in multiple physiological disturbances, including sympathetic nervous system activation, increased energy cost of breathing, intermittent hypoxemia, and wide swings in intrathoracic pressure. This disorder is highly prevalent in the general population, affecting more than 10% of middle-aged adults, with increased prevalence observed with aging, obesity, and cardiometabolic disease, and is more common in men [8]. OSA leads to sleep disruption, particularly increased sleep fragmentation and decreased proportion of restorative stages of sleep, resulting in daytime sleepiness, impaired quality of life and cognitive deficits [9]. Moreover, OSA is associated with increased rates of hypertension, incident heart disease, stroke, diabetes, depression, certain cancers, and overall mortality [10–19]. Despite the large number of epidemiological studies indicating that OSA is closely associated with these outcomes, there appear to be subgroup differences in susceptibility, e.g., middle-aged individuals and men are more likely to experience OSA-related cardiovascular disease in some studies than older individuals and women, respectively [20]. This underscores gaps in our knowledge of the pathophysiological pathways linking OSA to other diseases [21,22]. Pathophysiological pathways linking OSA to other diseases and factors that influence individual differences in susceptibility are poorly understood. While there are several effective treatments for OSA, including Continuous Positive Airway Pressure (CPAP), there appears to be substantial variation in overall clinical response and attenuation of cardiometabolic consequences, suggesting heterogeneity in both the etiology of the disease and susceptibility to its physiological disturbances.

Indices of OSA, including the Apnea-Hypopnea Index (AHI; the number of breathing pauses per hour of sleep), apnea event duration, indices of overnight hypoxemia, habitual snoring, and excessive daytime sleepiness, show substantial heritability in family studies [23]. Past studies have identified only a handful of associations with a variety of OSA-related traits. We have previously described a GWAS of OSA traits measured by overnight polysomnography in multi-ethnic cohorts totaling ~20,000 individuals [24]. In that study, we found two genome-wide significant multiethnic associations: variants in a locus on 10q22 were associated with indices of average and minimum SpO_2_ and percentage of sleep with SpO_2_ < 90%, and variants in a locus on 2q12 were associated with minimum oxygen saturation (SpO_2_). In another study, we identified a locus in 17p11 with a male-specific effect on AHI [25]. Furthermore, in an admixture mapping study in Hispanic/Latino Americans, we identified a locus on 2q37 associated with AHI and one in a locus on 18q21 associated with AHI and SpO_2_ < 90% [26].

The low number of genetic associations reported to date only explains a small fraction of OSA trait heritability. This relative paucity of findings is driven primarily by modest sample sizes, a reflection of the expense and complexity of measuring physiological phenotypes by overnight polysomnography or respiratory polygraphy. This also limits our ability to fine-map associations down to causative variants and thus identify relevant genes. Data on hundreds of thousands of individuals–sample sizes at which GWAS designs are well-powered to detect tens of loci and, in combination with additional experiments, fine-map some of them–have yet to be collected for OSA traits based on overnight polysomnography or respiratory polygraphy and may never be available [1,2]. Biological interpretation of available genetic associations is further complicated by the observation that most GWAS effects localize to enhancer regions and other regulatory elements and are often distal to physiologically relevant genes [27,28].

Now that GWAS of massive sample sizes have been accumulated for various comorbid conditions and endophenotypes related to OSA, we hypothesize that analysis of shared associations across correlated traits can identify effects in underpowered studies of OSA and generate clear and testable biological hypotheses. A number of computational methods increase power for discovering genetic associations by capitalizing on pleiotropy between disease phenotypes or between a disease and a molecular trait such as gene expression. One common approach takes advantage of the genetic correlation among phenotypes [6,29–31]. This class of methods gains substantial additional power by pooling association signals across traits. However, such methods suffer from power loss when the correlation of genetic effect sizes is highly variable across the genome or limited to a subset of loci. For example, an approach such as MTAG is not suitable for small GWAS studies when the assumption of the homogeneity of genetic correlation is violated. The estimated genetic effect of an underpowered trait can be inflated by the strong genetic signals of well-powered traits that are pooled together even if the variant is not causative for the underpowered trait [6]. An alternative approach analyzes individual loci to detect pleiotropic alleles, with no regard to genetic correlation [4,7,32–41]. Only a handful of existing methods account for the possibility that the apparent pleiotropy is driven by the linkage disequilibrium (LD) between two distinct causative variants each of which drives only one phenotype [4,36–40].

In this study, we apply a colocalization method to detect shared associations between OSA and other related traits. We elected Joint Likelihood Mapping method (JLIM) [39] and eCAVIAR [38] as colocalization methods, but our general strategy does not depend on any specific method. We focus on a set of well powered intermediate traits which have previously been implicated in the pathobiology of OSA. Given prior GWAS studies suggesting the involvement of inflammatory genes in OSA [42–44], and cohort studies reporting high levels of inflammation, including elevations in neutrophils and monocytes in OSA [45,46], we included leukocyte and platelet related traits in our pleiotropic comparisons. Similarly, we also included red blood cell related traits given prior GWAS implicating iron metabolism [26] and erythrocyte function [24]. We will refer to these as clinical traits. In addition, OSA is associated with lung [47,48], obesity and cardiovascular-related disorders [46,49–51], and we have included clinical traits that reflect these overlaps, together with gene expression traits in tissues implicated in these diseases.

By linking different clinical and gene expression traits to OSA at specific loci, our analysis suggests new hypotheses about OSA pathobiology across many physiological layers as well as finding a new association.

## Results

### Creating a framework to identify associations in underpowered GWAS through pleiotropy

We used a colocalization method to identify pleiotropic loci, where a genetic effect drives association to two traits. First, we selected genome-wide significant loci (association *p* < 5 x 10^−8^) in our well-powered trait (here, a clinical trait), and from these we selected the subsets which also show nominal association to OSA traits (*p* < 0.01 for any SNP in the locus). We then used colocalization to directly evaluate if the association to the two traits was consistent with the same underlying effect, indicating a pleiotropic effect. This two-step strategy allowed us to distinguish between cases where there was association only in the clinical trait; where there was a shared association in both traits; and if there were distinct associations in both traits stemming from different underlying effects (S1 and S2 Figs).

### Simulations

We first used simulations to assess our strategy to identify true associations in an underpowered study by detecting pleiotropic associations with a better-powered study of another trait and following up with replication in an independent cohort. To assess sensitivity and specificity, we simulated variable proportions of shared and distinct causal effects, whilst assuming high polygenicity for each trait and only one causative variant per locus. We simulated GWAS statistics for pairs of traits corresponding to our own study design: we simulated situations where no variant is causative for an underpowered trait (H_0_), where the same variant is causative for the association to both traits (H_1_), and where two distinct variants in LD drive each of the associations at a locus (H_2_) (Methods). To simulate summary statistics from a well-powered GWAS (representing our clinical traits), we sampled values from a multivariate normal (MVN) distribution using the local LD matrix as a variance-covariance parameter [52] with clinical trait sample size of 150,000. To simulate a GWAS of limited power (representing our OSA discovery cohorts), we used genotypes from ancestry-matched samples as a reference from which we simulated quantitative traits in 10,000 samples as surrogates for the OSA GWAS. Most overlapping associations are expected to be due to distinct variants [39]; we simulated scenarios with just 5% and 20% of true associations being driven by the same variant in both traits (H_1_). For example, in the first case, we simulated 2,500 loci with 3.5% corresponding to H_1_, 66.5% corresponding to H_2_ and 30% corresponding to no association in the underpowered trait (H_0_). The focus of our simulation study was the ability to replicate the association signal for the primary phenotype in an independent cohort. This means successfully discriminating between (H_1_+H_2_) and H_0_. We leave the interpretability question (discriminating H_1_ from H_2_) to Discussion.

**Fig 1:**
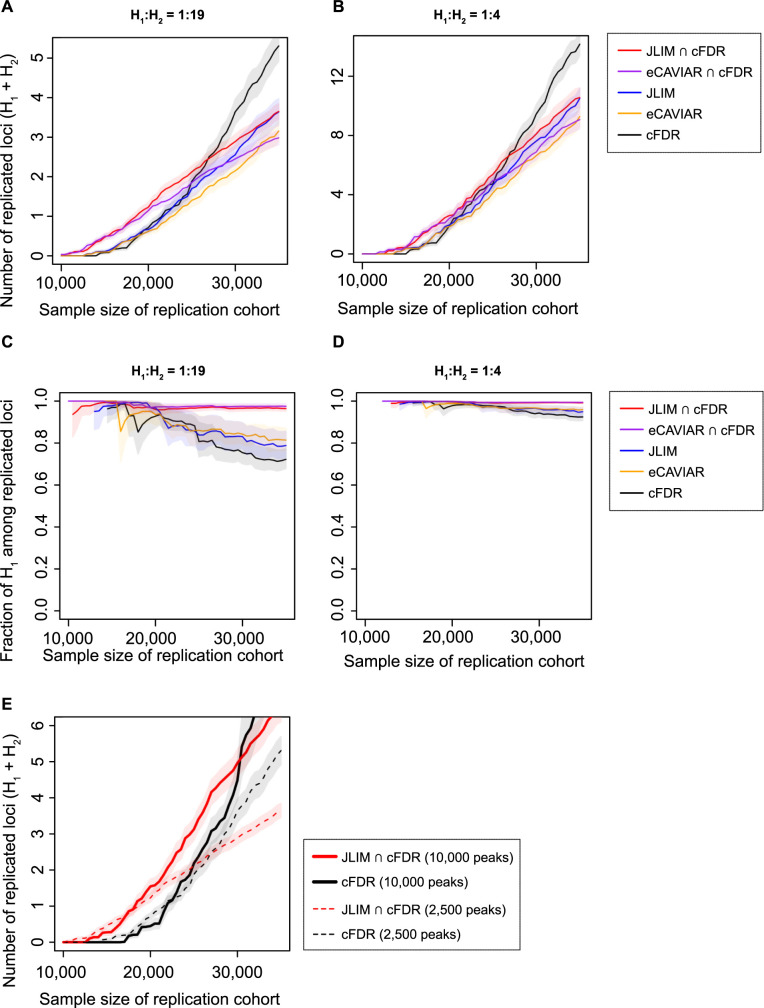
The projected number of replicated loci and proportion of shared causative variants among the replicated loci. A total of 2,500 association peaks from well-powered GWAS studies (n = 150,000) were tested for the shared effect in simulated discovery cohorts (n = 10,000), and then select loci were tested for replication in simulated validation cohorts of the same genetic ancestry (n = 10,000–35,000). The candidate loci were identified by conditional false discovery rate (cFDR), colocalization tests (JLIM and eCAVIAR), or the intersection of cFDR and colocalization test. The *p*-value cutoff was set to 0.01 for cFDR and JLIM, and for eCAVIAR, the equivalent posterior threshold was calibrated using H_0_ simulation data. The 2,500 GWAS peaks consist of the loci simulating no causal effect for underpowered traits (H_0_) and those simulating the same causal effect between two traits (H_1_) or distinct causal effects (H_2_). The H_1_:H_2_ ratio was set to 1:19 (**A** and **C**) or 1:4 (**B** and **D**). The effect sizes of causative variants are correlated (*ρ* = 0.7) under H_1_ but uncorrelated under H_2_. Bonferroni correction was applied on replication tests. In Panel **E**, we contrast the number of replicated loci expected in Panel **A** (dashed line) with a more extreme scenario (10,000 GWAS peaks, consisting of 2,500 with H_1_:H_2_ of 1:19 and 7,500 with H_1_:H_2_ of 1:39; solid lines). In all, the proportion of H_0_ was set to 30% of all examined loci. The shaded area denotes the 95% CIs.

We tested two colocalization methods–JLIM and eCAVIAR (a popular Bayesian colocalization test) [38,39]. We also tested a popular pleiotropy-informed test, conditional false discovery rate (cFDR) [32,33]. cFDR is not based on the LD-informed colocalization of the association signals. All methods appear underpowered in the discovery dataset at a sample size typical of current genetic studies of OSA (n = 10,000); the methods are only able to find associations if the observed effect is larger than the true effect (S3 and S4A Figs). Among the true associations identified by JLIM, eCAVIAR and cFDR, there is an enrichment of H_1_ loci. In spite of this enrichment, many loci identified in the discovery cohort are driven by distinct variants (H_2_): (for the simulation with 5% of the true associations being H_1_, 54.7, 55.8 and 61.5% of loci identified by JLIM, eCAVIAR and cFDR, respectively, are H_2_). At the same time, almost all proportions of successfully replicated associations are driven by the same variant (H_1_) in our simulation regimes (Figs 1C and 1D and S4B–S4F).

Because the colocalization tests (such as JLIM and eCAVIAR) and cFDR are using different features of the data, we found that taking a consensus between them can effectively exclude the majority of H_2_ from the analysis (S3 Fig). In the intersection of JLIM and cFDR, only 3.5% of identified loci are H_2_; in the intersection of eCAVIAR and cFDR, only 5.3% are H_2_ (S4A Fig). Even though the elimination of H_2_ loci increases the false discovery rate (fraction of H_0_) in the discovery sample, it substantially increases the rate of successful replication. The main reason for the increase in the rate of successful replication is the prioritization of H_1_ loci of larger true effect size and the corresponding reduction of multiple testing burden at the replication stage. The benefit of this approach increases with the fraction of loci driven by distinct effects (H_2_) (S5 Fig). It also increased when the fraction of loci of no true effect (H_0_) is low (as in the case of very high polygenicity [53] (S6 Fig)).

To test if the consensus method allows for more replicated loci simply due to the smaller number of candidate loci, we tightened the *p*-value threshold of cFDR to select the same number of candidates in discovery as the consensus method (S7 Fig) or to control the rate of H_0_ loci (S8 Fig). We also explored a range of other simulation conditions, including varying the total number of examined well-powered trait-associated loci, correlation of effect sizes between well-powered and underpowered traits, trans-ethnic validation cohort, and the presence of multiple causative variants in locus (S9–S12 Figs). Consistently, the consensus method resulted in more replicated loci than cFDR alone when the sample size is limited.

Next, we compared the rate of successful replication between the consensus approach and Bayesian meta-analysis methods. In contrast to colocalization methods, the Bayesian meta-analysis approach leverages the correlation of effect sizes between traits rather than SNP-level colocalization of causative variants [29–31]. We chose to compare two such Bayesian meta-analysis methods, MetABF and CPBayes, with the consensus approach using the simulated dataset. Again, when the sample size was limited (n < 20,000), our consensus method found more replicated associations than meta-analysis approaches (S13 and S14 Figs). As the sample size of the validation cohort increases, both MetABF and CPBayes identified more H_2_ loci, which led to more replicated loci than the consensus method. Since the meta-analysis methods are designed for a pleiotropy analysis of more than two traits, in principle, they can boost the power by using more than one well-powered trait. To test if the multi-trait meta-analysis can outperform the pairwise meta-analysis, we simulated nine additional well-powered traits, generating GWAS statistics for one underpowered trait and ten well-powered traits in each locus (Methods). For the additional well-powered traits, we randomly decided whether the simulated causative variant was to be the same as or distinct from that of the main well-powered trait. Using this simulated dataset, we examined the power of the above Bayesian methods and non-parametric meta-analysis (iGWAS) [7] in multi-trait and pairwise settings. Overall, we could not see a clear advantage of multi-trait meta-analyses over pairwise tests in our simulated data (S15 Fig). Rather, particularly for CPBayes and iGWAS, multi-trait tests substantially underperformed pairwise tests. This result demonstrates that for underpowered studies, the consensus approach focused on SNP-level colocalization outperforms meta-analysis techniques in pairwise and multi-trait settings when the association signals are driven by distinct rather than the same effect in a large fraction of loci.

Finally, we found that looking at more trait combinations with more GWAS peaks results in proportionally more discoveries (Fig 1E). Overall, our simulations demonstrate that casting a wide net across many traits (increasing the number of GWAS peaks) and taking the consensus between pleiotropy mapping methods is a viable strategy to increase discoveries in under-powered studies. We therefore felt justified in pursuing this strategy using real data, to make additional discoveries in traits related to OSA.

### Identifying pleiotropic associations between clinical traits and sleep apnea-related traits

Based on clinical relevance [54] and heritability [23], we focused on four OSA-related traits measured in five European-ancestry cohorts: the apnea-hypopnea index (AHI) [25], average respiratory event (apneas or hypopneas) duration [26], and minimum and average oxygen saturation (SpO_2_) during sleep [24]. We used summary statistics from the remaining multi-ethnic cohorts in our replication effort.

We assembled a collection of GWAS summary statistics for a total of 55 candidate intermediate traits from across these physiological areas: erythroid, leukocyte and platelet counts and function, from a study combining the UK Biobank and INTERVAL datasets (170,000 individuals of European ancestry) [55]; cardiovascular, metabolic and respiratory traits from the UK Biobank (380–450,000 European ancestry participants) [56,57], and cardio-metabolic traits (36,000 European ancestry participants) [58]. We then compared associations in each of these clinical traits to our OSA traits (6,781 European ancestry participants; S1 Table), to identify potential associations in the latter. A complete list of clinical traits we considered is presented in S2 Table.

We tested for directional causal effects of the selected clinical traits on our OSA related traits using Mendelian Randomization (MR) [59]. Due to the low sample sizes in OSA traits, no comparison reached statistical significance after multiple test correction (S3 Table). After excluding the extended MHC region and the sex chromosomes, we identified 3,191 genome-wide significant associations (*p* < 5 x 10^−8^) in the 55 clinical traits, of which 2,939 had a corresponding suggestive association to one of the four OSA traits (*p* < 0.01 at any SNP in the locus; S4 Table). We then explicitly tested for evidence of pleiotropy between clinical and OSA traits using JLIM (2,142 to 2,236 tests for each OSA trait). We found evidence that in 61/2,939 of these regions the OSA and clinical trait associations are consistent with a shared, pleiotropic underlying causative variant by JLIM (false discovery rate (FDR) < 0.20) and at the same time show evidence of association to OSA by cFDR (Tables 1 and S5).

**Table 1:** Loci with significant pleiotropic associations between a clinical trait and an OSA trait with nominally significant replication.

SNP	Coordinate	Clinical trait	Clinical trait *p*-value	OSA trait	OSA trait *p*-value	JLIM *p*-value	JLIM FDR	Replication *p*-value
rs17010961	4:86,723,103	Systolic blood pressure	7.9E-24	Avg O2 saturation	1.6E-05	1.4E-04	0.011	7.4E-03
rs2595105	4:111,552,761	Basal metabolic rate	7.0E-13	Min O2 saturation	1.0E-03	2.4E-03	0.177	0.036
rs4711750	6:43,757,082	Granulocyte % of myeloid white cells	6.7E-09	AHI	3.2E-03	9.0E-03	0.198	0.011
		Reticulocyte fraction of red cells	6.7E-11			9.0E-03	0.108	
		High light scatter reticulocyte % of red cells	5.4E-11			1.1E-02	0.191	
rs16926246	10:71,093,392	High cholesterol	2.8E-09	Min O2 saturation	1.1E-05	3.0E-03	0.051	0.039
				Avg O2 saturation	3.5E-05	1.0E-03	0.015	1.3E-03
rs17476364 *	10:71,094,504	Hematocrit	7.7E-159	AHI	7.3E-06	3.0E-05	0.001	0.016
		Reticulocyte count	1.9E-96			3.0E-05	0.001	
		Red blood cell count	1.8E-48			3.0E-05	0.002	
		Hematocrit	7.7E-159	Avg O2 saturation	3.9E-05	4.2E-04	0.017	**8.5E-05 †**
		Reticulocyte count	1.9E-96			4.2E-04	0.019	
		Red blood cell count	1.8E-48			3.8E-04	0.020	
		Hematocrit	7.7E-159	Min O2 saturation	3.1E-07	5.0E-05	0.002	0.014
		Reticulocyte count	1.9E-96			5.0E-05	0.002	
		Red blood cell count	1.8E-48			5.0E-05	0.003	
rs11245326	10:126,357,352	White blood cell count	4.3E-09	Event Duration	9.2E-05	2.2E-03	0.057	0.017
rs2277339	12:57,146,069	Plateletcrit	1.1E-10	AHI	5.2E-03	1.0E-02	0.154	**8.2E-04 †**
rs2297066	14:103,566,835	Platelet distribution width	2.4E-23	AHI	1.8E-03	3.2E-04	0.015	0.011
		Mean platelet volume	7.2E-70			3.9E-04	0.025	
		Platelet count	4.8E-39			1.0E-03	0.064	
rs998584	6:43,757,896	Reticulocyte count	2.5E-13	AHI	1.6E-02	1.3E-02	0.133	0.016
rs765807623	14:103,571,697	Plateletcrit	7.0E-09	AHI	1.8E-03	3.8E-04	0.027	0.011

Each row denotes one SNP and its corresponding associations to clinical and OSA traits. Each SNP may be associated with more than one clinical trait. At SNPs marked with *, not all clinical traits are shown for clarity. Here, we only show variants with nominally significant replication p-values. See S5 Table for the full table of 61 loci (137 trait pairs), including those with insignificant replication *p*-values. Two variants marked with † indicates pleiotropic loci with significant out of sample replication *p*-values after Bonferroni correction (0.05/61). AHI stands for Apnea-Hypopnea Index. Coordinates correspond to hg19. The clinical trait *p*-value column refers to the association *p*-value of the SNP to the clinical trait. The OSA trait *p*-value refers to the association *p*-value of the SNP to the OSA trait in meta-analyzed discovery cohorts (S1 Table). The replication *p*-value refers to the association *p*-value of the SNP to the OSA trait in meta-analyzed validation cohorts (S6 Table). The JLIM *p*-value corresponds to the tests for the shared effect between the clinical and OSA phenotypes.

**Fig 2:**
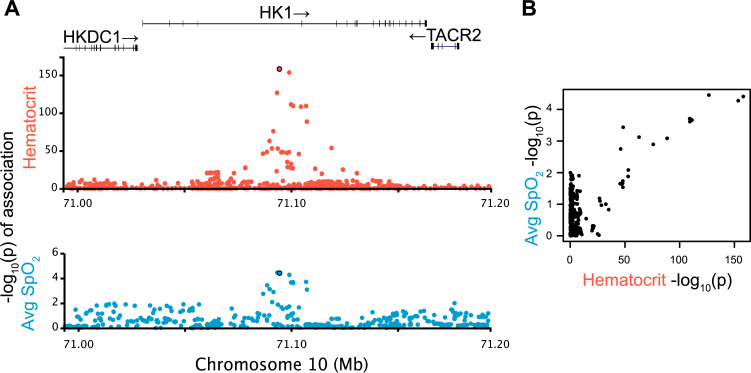
Pleiotropy at *HK1* locus. **A)** Putatively pleiotropic locus linking a clinical trait (hematocrit) with an OSA trait (average SpO_2_). The evidence of shared effect between the clinical and OSA traits is significant (JLIM *p* = 4.2 x 10^−4^, FDR = 0.017). This SpO_2_ association replicates in validation cohorts after Bonferroni correction of 61 tests (*p* = 8.5 x 10^−5^). **B)** Pairwise comparison of–log_10_(*p*-values) between two traits confirms that the association signals are driven by the shared underlying effect. Each dot represents a SNP in the tested locus.

To independently validate our 61 putative OSA trait associations from the discovery stage, we compiled summary statistics for the same traits in 15,594 individuals of Asian, African, European and Hispanic ancestries/backgrounds (S6 Table). These individuals do not overlap with those from the cohorts used in our colocalization analysis using JLIM. We did not attempt to replicate pleiotropic associations; we only replicated the OSA association statistics. JLIM relies on local LD patterns being preserved between clinical and OSA trait cohorts, so we cannot use multi-ethnic data in our discovery analysis of pleiotropy. We found that 2/61 variants in S5 Table show significant association with the same OSA trait as the initial observation, after Bonferroni correction for the number of tests performed. The variant in SNP rs17476364 (Fig 2) links 11 of 12 red blood cell related clinical traits analyzed with average SpO_2_ during sleep. It is an intronic variant in the hexokinase 1 (*HK1*) region in chromosome 10, and has been previously reported, as it reached genome-wide significance in association to minimum and average SpO_2_ [24]. Another variant in SNP rs2277339 is a missense coding variant in DNA primase subunit 1 (*PRIM1*) in chromosome 12. It links plateletcrit (ratio of platelet volume to whole blood volume, a marker of thrombotic as well as a broad range of inflammatory processes [60]) to AHI. In the UK Biobank, it has documented significant associations to height, waist to hip-ratio, age at menopause and multiple red blood cell related traits [61]. Further 8 variants, shown in Table 1, were below nominal association *p* values < 0.05 in the replication set, but did not survive multiple test correction.

### Incorporating gene expression to construct molecular hypotheses of sleep apnea physiology

Non-coding regions with evidence of gene regulatory activity carry a large proportion of heritability in most traits analyzed in large GWAS [62]. We reasoned that some OSA causative variants would reside in such regulatory regions, and thus act on gene regulation. We therefore sought shared associations between gene expression traits and the clinical traits for which we identified a pleiotropic association in the 61 loci in Tables 1 and S5. To do so, we compiled expression quantitative trait loci (eQTL) data for protein-coding genes expressed in lung, liver, spleen and skeletal muscle from individuals with European ancestry from the GTEx Project [63], and monocyte, T cell and neutrophil populations in individuals from BLUEPRINT [64]. We chose these tissues for potential relevance to OSA pathology: the lung is involved in OSA-related hypoxemia [47,48]; previous GWAS associations have implicated the neuromuscular junction in overnight SpO_2_ levels, and abnormalities in upper airway muscle function are fundamental mechanisms for sleep apnea [24,65]; the spleen and liver are known to mediate filtration of erythrocytes, iron homeostasis and production of inflammatory cytokines; and leukocytes are key modulators of inflammation, an antecedent risk factor of OSA development [66]. We calculated FDR based on JLIM *p*-values over the 5,860 comparisons against 1,009 protein-coding genes of which transcription start sites are within 1 Mb from the index SNPs of clinical traits.

**Fig 3:**
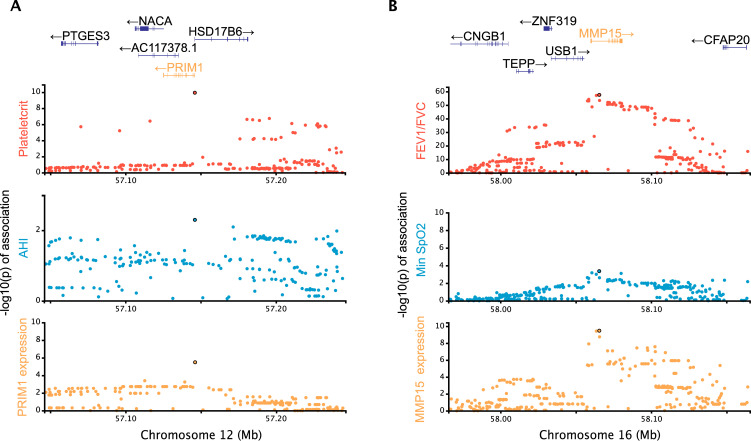
Candidate causal chains linking a clinical trait, OSA trait and gene expression. **A)** A candidate association in chromosome 12 with putative pleiotropic associations between the plateletcrit (clinical trait; red), AHI (OSA trait; blue) and expression of *PRIM1* (DNA primase subunit 1) in monocytes (yellow). **B)** A candidate association in chromosome 16 with putative pleiotropic associations between the clinical trait FEV_1_/FVC (red), minimum O_2_ saturation (blue) and expression of *MMP15* (matrix metallopeptidase 15) in lung (yellow).

We were able to identify shared associations between eQTL and clinical traits in 7/61 loci (FDR < 0.05; Table 2). This includes several notable examples, including rs2277339, one of our two replicated SNPs (Table 1). The rs2277339 SNP is a missense variant of *PRIM1* but also an eQTL for *PRIM1* levels in monocytes and T cells (Fig 3A). Another example is a locus on chromosome 16 where we find that an eQTL for *MMP15* (matrix metallopeptidase 15) expression in lung tissue is pleiotropic with a measure of lung function (FEV_1_/FVC, the ratio of forced expired volume per second to forced vital capacity), which in turn is pleiotropic with an association with minimum SpO_2_ during sleep (Fig 3B). The lead SNP for *MMP15* (rs4784886) is marginally associated with minimum SpO_2_ in the validation cohort (nominal *p* = 0.051). Overall, of the eight identified genes, four genes (*MMP15*, *SESN1*, *FOXO3* and *BRD3*) are known to be induced by hypoxia and oxidative stress, and three genes (*FOXO3*, *BRD3* and *GSDMA*) are implicated in inflammatory responses. The colocalization of associations to gene expression in specific cell types, clinical and sleep apnea traits suggests biological hypotheses of the pathophysiology underlying OSA.

**Table 2:** Candidate causal chains.

SNP	Coordinate	Clinical trait	OSA trait	JLIM *p*-value	JLIM FDR	Tissue / Cell type	Gene	eQTL *p*-value	JLIM *p*-value	JLIM FDR
rs11153147	6:109,304,058	Mean corpuscular volume	Avg O2 saturation	1.1E-02	0.17	T-cell	*SESN1* (Sestrin 1)	1.4E-35	5.0E-05	0.01
						Muscle	*SESN1* (Sestrin 1)	2.6E-05	5.0E-05	0.05
						T-cell	*FOXO3* (Forkhead box O3)	1.7E-11	5.0E-05	0.01
rs4784886	16:58,065,459	FEV1/FVC	Min O2 saturation	3.4E-04	0.03	Lung	*MMP15* (Matrix metallopeptidase 15)	3.3E-10	5.0E-05	0.02
rs10160596	11:65,351,364	Mean corpuscular hemoglobin concentration	Event Duration	1.0E-02	0.17	Lung	*SCYL1* (SCY1 like pseudokinase 1)	5.1E-04	5.0E-05	0.02
rs146671954	9:136,934,203	Red cell distribution width / Sum eosinophil basophil counts	AHI	6.9E-04	0.02	Lung	*BRD3* (Bromodomain containing 3)	2.2E-07	1.0E-04	0.02
rs2277339	12:57,146,069	Plateletcrit	AHI	1.0E-02	0.15	monocyte	PRIM1 (DNA primase subunit 1)	3.2E-06	5.0E-05	0.04
						T-cell	PRIM1 (DNA primase subunit 1)	1.5E-05	5.0E-05	0.01
rs7162943	15:89,615,275	Mean platelet volume	Avg O2 saturation	1.0E-03	0.07	monocyte	*ABHD2* (Abhydrolase domain containing 2, acylglycerol lipase)	2.9E-15	1.0E-04	0.04
rs34233420	17:38,004,929	Lymphocyte count	AHI/ Min O2 saturation	3.4E-04	0.02	T-cell	*GSDMA* (Gasdermin A)	2.5E-13	5.0E-05	0.01

Each row represents a SNP with links across an OSA trait, clinical trait and gene expression trait. The link between clinical and OSA traits were thresholded at FDR < 0.2, and the link between clinical and expression traits were thresholded at FDR < 0.05. AHI stands for Apnea-Hypopnea Index. Coordinates correspond to hg19. The eQTL *p*-value refers to the association *p*-value of the SNP to the gene expression trait of the gene in the tissue/cell type indicated. The JLIM *p*-value corresponds to the test for the shared effect between the OSA and clinical traits (center columns) or between gene expression and the clinical trait (right columns).

**Fig 4:**
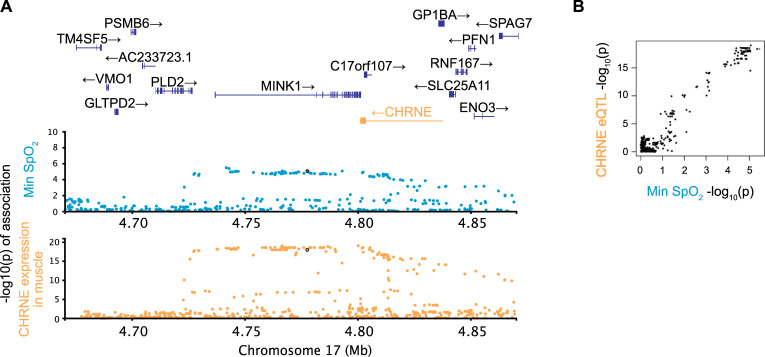
Pleiotropic locus linking gene expression and an OSA related trait. A locus in chromosome 17 has associations between minimum oxygen saturation (blue) and expression of *CHRNE* (cholinergic receptor nicotinic epsilon subunit) in muscle tissues (yellow). Gene expression trait *p*-values and the appropriate gene in the locus are shown in yellow. Pairwise comparisons of–log_10_(*p*-values) between associated traits are shown in Panel **B** with matching colors in axis labels.

We also investigated pleiotropy between gene expression traits and OSA in the three loci harboring known genome-wide significant OSA associations in the discovery sample (Tables 3 and S7). In each locus, we compared OSA trait summary statistics to eQTLs for genes within 1Mb from the most associated variant, where there exists a SNP with eQTL association *p*-value < 5x10^-8^ in the locus. We replicated a previously found pleiotropic effect in a locus on chromosome 17 [24], where minimum oxygen saturation (SpO_2_) colocalizes with expression of the epsilon subunit of the nicotinic receptor (*CHRNE*), in various tissues, including neutrophils, monocytes, spleen and muscle (Fig 4).

**Table 3:** Genome-wide significant loci in OSA traits colocalizing with eQTL.

SNP	Coordinate	OSA trait	OSA assoc *p*-value	Gene	Tissue / cell type	eQTL *p*-value	JLIM *p*-value	JLIM FDR
rs12150370	17:4,777,634	Min O_2_ Saturation	9.0E-06	*CHRNE* (cholinergic receptor nicotinic epsilon subunit)	Muscle	9.3E-20	2.0E-05	0.0006
					Neutrophil	2.5E-20	2.0E-05	0.0006
					Spleen	3.4E-13	8.0E-05	0.001
					Monocyte	8.8E-14	9.0E-05	0.001
				*C17orf107* (chromosome 17 open reading frame 107)	Spleen	1.4E-08	5.0E-05	0.001

Three loci with known genome-wide significant association to OSA traits were tested for the shared effect with gene expression levels in seven tissue/cell types. The FDR cutoff of 0.05 was applied. The eQTL *p*-value refers to the association *p*-value of the SNP to the gene expression trait of the gene indicated, measured in the tissue/cell type indicated. The JLIM *p*-value refers to a pleiotropy test between the OSA and gene expression traits. See S7 Table for the full list of comparisons tested.

## Discussion

In our comparison of clinical (respiratory, cardiometabolic, hematologic, inflammatory) traits to OSA-related traits, the strongest finding lies in an intronic region of hexokinase 1 (*HK1*) and is associated with average overnight oxygen saturation level (SpO_2_). This locus is pleiotropic with most of the red blood cell related traits tested (Fig 2) and corresponds to one of the most significant genome-wide associations we had previously reported from this data [24]. Prior to this analysis, two alternative hypotheses for the etiology of this signal had been proposed: that *HK1* acted by modulating inflammation, or that it affected OSA by altering erythrocyte function. Our results provide evidence that is consistent with the erythrocyte pathway hypothesis. Mutations in *HK1* have been implicated in anemia, together with severe hemolysis and marked decreases in red blood cells [67]. As discussed previously [24], it is possible that *HK1* affects the Rapoport-Luebering shunt through glycolytic pathway intermediates, which in turn mediates oxygen carrying in mature erythrocytes. Factors that influence arterial oxygen levels can lead to a more severe OSA phenotype (i.e., lower average levels of oxygen saturation predispose to greater hypoxemia with each breathing obstruction). Lowered oxygen carrying capacity and thus more tissue hypoxia could also contribute to breathing instability (and thus apneas) via Hypoxia-Inducible Factor-1 (HIF-1) and enhanced carotid body sensitivity and chemoreflex activation, or through long-term respiratory facilitation and plasticity [68,69].

The analysis of pleiotropy can be used to concatenate more than one phenotype to create candidate “causal chains,” which by linking eQTLs to well-powered traits to sleep apnea related traits can hint at promising biological targets. Among the most significant results for this multicomponent model is DNA primase subunit 1 (*PRIM1*). We found that a missense variant in *PRIM1* colocalizes with its own expression levels in monocytes and T cells, plateletcrit and AHI. The association of this variant with AHI was replicated in an independent validation cohort. The *PRIM1* deficiency is known to cause lymphopenia along with severe growth retardation, microcephaly and “triangular face” [70]. The impact of *PRIM1* on craniofacial morphology may contribute to OSA by causing a narrow airway, and thereby increasing the risk of airway obstruction during sleep; however, an inflammatory mechanism is also possible.

Another example of causal chains is matrix metallopeptidase 15 (*MMP15*), a gene whose expression in lung tissue is affected by an eQTL that colocalizes with a lung function phenotype (FEV_1_/FVC) which itself colocalizes with minimum SpO_2_ during sleep. Matrix metallopeptidases (MMPs), a family of proteolytic enzymes that can be activated by inflammation and oxidative stress, participate in and potentiate tissue remodeling by breaking down the extracellular matrix. MMPs have been suggested to play an etiological role in OSA-related pathophysiological responses that may lead to multiple organ dysfunction [71], and specifically, may lead to OSA by contributing to abnormalities in the extracellular matrix of the skeletal muscle in the upper airway, predisposing to passive airway collapse [72]. In particular, *MMP15* (membrane-type 2 MMP) is known to be upregulated by HIF-1α under hypoxic conditions [73,74] and is expressed in alveolar epithelial cells in Idiopathic Pulmonary Fibrosis (IPF) [75], an Interstitial Lung Disease (ILD) characterized by chronic inflammation, progressive formation of scar tissue and decreased lung function. OSA is highly prevalent in ILD as well as associated with subclinical markers of ILD [47]. OSA is also common in Chronic Obstructive Pulmonary Disease (COPD), and this overlap is especially associated with more severe hypoxemia [76]. Our results suggest a common causal pathway linking these lung diseases with OSA.

We also found that *GSDMA* (Gasdermin A) eQTL in T cells colocalizes with associations to the lymphocyte count, AHI as well as oxygen saturation. While Gasdermin A is considered to be expressed mostly in epithelial cells rather than in T cells, broadly, gasdermins mediate inflammatory responses via pyroptosis, a form of programmed cell death leading to the release of proinflammatory molecules, and play a key role in NLRP-inflammasome responses. Increased level of gasdermin D has been shown to mediate hypoxia-related muscle injury in an animal model of OSA [77]. Inflammatory mechanisms may increase risk for OSA through effects on soft tissue structures in the upper airway, muscle function, and/or neural control of breathing, as suggested by a prospective study of inflammation leading to higher risk of OSA [66].

Another interesting result is an eQTL in the epsilon subunit of the nicotinic acetylcholine receptor *CHRNE* that colocalizes with a genome-wide significant association in minimum oxygen saturation. This receptor is present at neuromuscular junctions, and mutations in this subunit are known to cause congenital myasthenic syndrome in humans that can result in progressive respiratory impairment [78]. As discussed above, abnormal skeletal muscle responses in the upper airway are considered to be central in the pathogenesis of OSA.

We have shown that we can leverage pleiotropy between OSA and physiological traits to identify the known OSA association in the *HK1* locus. We could not identify two other known OSA associations at 2q12 and 17p11 using this approach, as there is no apparent pleiotropy between OSA and our library of clinical traits. Adding gene expression traits to this analysis increases our discovery ability, as we can identify the 17p11 association as pleiotropic with an eQTL for *CHRNE* in neutrophils, monocytes, spleen and muscle (Table 3). Furthermore, in simulations, we showed that using a large array of clinical trait-associated loci has a potential to boost the power to identify associations in underpowered studies by casting a wider net (Fig 1E). These results warrant further investigation on using gene expressions in diverse cell types and conditions as a library of well-powered traits for underpowered association studies.

Replication in an independent cohort is frequently necessary to validate GWAS findings in small discovery samples. In this study, we significantly replicate the association of two loci to OSA after Bonferroni correction and identify additional eight loci that replicate only nominally but are probably enriched with true positive associations to OSA (S16 Fig). We randomly selected 28,558 unlinked autosomal SNPs (*r*^*2*^ < 0.1 within 100kb windows) from our initial OSA GWAS in individuals of European ancestry. We focused on AHI since we detected the most SNPs reaching nominal replication in this trait. We found that 11,910 of these would have been selected for our colocalization analysis, in line with expectation for the presence of a SNP within the 100kb distance satisfying our in-sample threshold of association *p* < 0.01. Out of these, 6,123 had a significant out-of-sample nominal replication (association *p* < 0.05). In comparison, our pleiotropy analysis results for AHI are 4.6-fold enriched at this threshold (4/20 independent hits with *r*^*2*^ < 0.1), suggesting the presence of true associations to sleep traits (one-sided Fisher Exact Test *p* = 0.018). In fact, the 76/975 associations that did not show significant evidence of pleiotropy were also slightly enriched for nominal replication relative to the set of randomly selected variants (one-sided Fisher Exact Test *p* = 0.00048, 1.6-fold enrichment). This suggests that additional pleiotropic effects–and therefore true OSA associations–remain to be discovered above the FDR cutoff we applied to our colocalization results although we cannot rule out the possibility that the random SNPs may not fully recapitulate the functional properties of GWAS SNPs from clinical traits.

From a methodological perspective, the analysis of pleiotropy has become an important tool in the analysis of complex trait genetics. Most complex traits are highly polygenic, implying that many variants associated with a single trait will also be associated with other traits or will be in LD with such variants. Different computational methods are required for different applications and for different genetic architectures. If the goal is to increase power to detect an association and the genetic correlation is broadly dispersed over many loci, methods explicitly capitalizing on the broad genetic correlation are capable of producing large power gains [6,29]. In cases where the majority of overlaps in GWAS association peaks between traits are driven by LD between distinct causative variants, power can still be increased with the help of other methods that leverage pleiotropy to reduce multiple testing burden [32,33]. Development of another group of approaches was motivated by the need to link genetic associations to genes via eQTL data [36,38,39] but, as shown here, these methods can be easily adapted to the analysis of other traits. Because of the abundance of association signals, especially for cellular and molecular traits, distinguishing between true pleiotropy due to the same underlying causative variants and different causative variants in LD is important for all the applications. Therefore, in our study of OSA, we selected a method that explicitly models LD structure in the locus. The drawback of this choice is the need to restrict the discovery sample to a demographically homogeneous subset while using the available multi-ethnic cohort for replication. While colocalization tests can distinguish the same and distinct causative variants in overall, the specificity is diminished in cases where association signals of two traits are driven by distinct causative variants in high LD (S2 Fig). For example, when the distinct causative variants are in LD between 0.8 and 0.95 (3.7% of simulated data assuming the random LD distribution), eCAVIAR and JLIM misclassify 1.8 and 2.3% of H_2_ loci, respectively, as driven by the same variant (JLIM *p* < 0.01 or at equivalent posterior probability threshold for eCAVIAR). JLIM estimates accurately calibrated *p*-values under H_0_ and conservatively approximate *p*-values under H_2_. For underpowered GWAS studies, with limited sample sizes, we lowered JLIM’s genetic resolution parameter *θ* to 0.5 in this study. JLIM does not attempt to distinguish distinct causative variants beyond its specified genetic resolution from the same causal effect. When distinct causative variants for two traits are separated with LD *r*^*2*^ < 0.5, JLIM maintains the ability to distinguish the same and distinct effects in simulations (S2D–S2F Fig). And the overall specificity of JLIM to distinguish H_1_ from H_2_ is similar or higher compared to other colocalization method such as eCAVIAR in our simulated datasets (S2B Fig).

Pleiotropy does not necessarily imply a causal relationship between phenotypes. Confounding by unadjusted covariates in GWAS data can further complicate the interpretation of causal chains due to the difficulty to distinguish between direct and indirect associations. Nonetheless, as we demonstrate here, a shared genetic basis between OSA and organismal, cellular and molecular traits can reveal new aspects of the underlying biology. This will likely be of benefit for other clinically relevant traits that are difficult to study at the scale required for GWAS. Traits that are burdensome or expensive to phenotype, rare diseases that are hard to sample and diseases that affect under-represented populations could all lead to underpowered genetic studies, which are unlikely to get dramatically higher sample sizes in the near future. Therefore, there is an unmet need to optimize the signals that can be extracted from small GWAS, and the strategy presented here should help in achieving this goal.

## Materials and methods

### Ethics statement

This research was approved by Partners Healthcare IRB (protocol #2010P001765).

### OSA Cohorts

To study pleiotropic associations underlying the risk of OSA, we prepared two sets of cohorts: the discovery cohorts to identify pleiotropic variants and independent replication cohorts to validate their associations to OSA traits. For the discovery cohorts, we used individual-level genotype data in order to determine the significance of pleiotropy by permutation (JLIM) [39]. At the replication stage, we do not carry out any pleiotropy analysis, we only check for genetic association to OSA, so summary-level association statistics were sufficient. In addition, we restricted the genetic ancestry of GWAS discovery cohorts to that of European ancestry, to match GWAS of clinical traits. This was in order to avoid potential issues due to the mismatch of LD patterns in our pleiotropy analysis. In contrast, we did not require the replication cohorts to have any specific ancestry. Thus, our replication cohorts included all available ethnicities.

The discovery cohorts included the subset of samples of European ancestry from the following five cohorts: the Atherosclerosis Risk in Communities Study (ARIC) [79], Osteoporotic Fractures in Men (MrOS) Study [80], Multi-Ethnic Study of Atherosclerosis (MESA) [81], Cardiovascular Health Study (CHS) [82] and the Western Australian Sleep Health Study (WASHS) [83]. ARIC is a study that investigates atherosclerosis and cardiovascular risk factors. It is one of the cohorts included in the Sleep Heart Health Study, which collected polysomnography and genotype data [84]. Genotype data were obtained through dbGaP (phg000035.v1.p1). MESA is a population-based study focused on cardiovascular risk factors, which included participants of four ethnicities: African-, Asian-, European- and Hispanic/Latino-Americans ranging from ages of 45 to 86 years old. We only included samples from European-Americans in the discovery cohort. Polysomnography data measuring sleep-related traits were later obtained from individuals who did not use overnight oxygen, CPAP or an oral device for sleep apnea [85]. MrOS is a multi-center prospective epidemiological cohort assembled to examine osteoporosis, fractures and prostate cancer in older males [86]. An ancillary study (MrOS Sleep) measured sleep disturbances and related outcomes [87]. CHS is a cohort aimed to study coronary heart disease and stroke in individuals aged 65 and older, and genotype data were obtained through dbGaP (Illumina CNV370 and IBC; phg000135.v1.p1 and phg000077.v1.p1). WASHS is a clinic-based study designed to examine OSA and its associated genetic risk factors in patients referred to a sleep clinic in Western Australia. Not all individuals had measurements for the four OSA-related traits of interest. Details on genotyping, imputation and QC procedures have been previously reported [24]. See S1 Table for the details.

The replication cohorts were: the Hispanic Community Health Study/Study of Latinos (HCHS/SOL) [88,89], Starr County Health Studies (Starr) [90], Cleveland Family Study (CFS) [91] and Framingham Heart Study (FHS) [92], in addition to non-European samples of CHS and MESA. HCHS/SOL is a population-based study to examine protective and risk factors for many health conditions among Hispanic/Latinos living in four urban areas within the USA (Chicago IL, Miami FL, San Diego CA and Bronx NY). Starr is a cohort collected to study risk factors for diabetes in a population of Mexican-Americans in Texas, later phenotyped for sleep traits [93]. CFS is a family-based study, which recruited patients with OSA, their relatives and neighborhood control families to study the familial and genetic basis of sleep apnea (356 families of African American or European American ancestry). We included only unrelated individuals from CFS. FHS is an epidemiological cohort established to study cardiovascular disease risk factors, using follow-up medical examinations every two years for the population of European Ancestry in Framingham, MA. Data from the first Sleep Heart Health Study were obtained between 1994–1998. Genotype data were obtained through dbGaP (Affymetrix 500k; phg000006.v7). See S6 Table for the details of each cohort.

We examined the following four OSA-related traits in the discovery and replication cohorts: minimum and average oxygen saturation (SpO_2_), apnea-hypopnea index (AHI) and event duration. Briefly, the minimum and average SpO_2_ were calculated from oximetry-based SpO_2_ measurements over the entire recorded sleep interval excluding occasional waking periods. AHI was scored by counting the number of episodes of complete (apnea) or partial (hypopneas) airflow reduction associated with ≥ 3% desaturation per hour of sleep. The event duration was measured for the average length of apneas and hypopneas, from the nadir of the first reduced breath to the nadir following the last reduced breath (in seconds). The full description of phenotyping protocols is present in the original studies which first reported their genetic analysis in the context of OSA [24–26]. We rank-normalized all OSA traits, separately in each cohort, in order to obtain normally distributed phenotypes.

### Clinical trait data

For clinical traits, we used GWAS summary statistics calculated for various traits in the UK Biobank [56,94], blood cell-related phenotypes in a general UK population [55] and cardio-metabolic phenotypes in individuals of European ancestry [58]. There is no sample overlap between clinical trait GWAS data and our discovery or replication cohorts. The full list of clinical traits is shown in S2 Table. The GWAS summary statistics for UK Biobank traits and blood cell counts were downloaded from their websites. The summary statistics of cardio-metabolic traits from [58] were obtained directly from the authors.

### Identifying pleiotropic variants affecting both clinical traits and OSA

We applied Joint Likelihood Mapping (JLIM version 2.0) [39] to test whether the association signals of clinical and OSA traits were driven by a shared genetic effect. We ran JLIM only on the loci in which there was strong evidence of association to a clinical trait (genome-wide significant) and a suggestive association to the OSA trait (*p* < 0.01 at any SNP in the locus). In these loci, JLIM compares the likelihood of observed association signals under the following three competing possibilities: the OSA trait has no causative variant in the locus (“H_0_”), the same OSA causative variant is shared with the clinical trait (“H_1_”), and the OSA causative variant is distinct from the clinical trait causative variant (“H_2_”), as shown in S1 Fig. Since underlying association data have the limited genetic resolution, we test for modified hypotheses $H_{1}^{\theta }$ and $H_{2}^{\theta }$ instead of H_1_ and H_2_. $H_{1}^{\theta }$ represents that the causative variants of two traits are identical or in high LD (*r*^2^>*θ*), and $H_{2}^{\theta }$ assumes that the causative variants are separated by the LD threshold (*r*^2^<*θ*). In our simulation of underpowered GWAS (see below for the details), only a small fraction (9.0%) of loci had lead SNPs in tight LD (*r*^2^>0.8) with causative SNPs; only 15.7% of loci had the modest LD of *r*^2^>0.5 between lead and causative SNPs. This contrasts with well-powered GWAS data, for which, in 91.4% of simulated loci, lead SNPs were in LD of *r*^2^>0.8 with causative SNPs. To account for this limited statistical resolution of underpowered GWAS data, we lowered *θ* to 0.5 from the default of 0.8 in this study.

JLIM calculates the ratio between the likelihood of the data under $H_{1}^{\theta }$ compared to that under $H_{2}^{\theta }$ and evaluates the significance of this statistic by permuting the phenotypes simulating the lack of causal effect under H_0_. The false positives due to $H_{2}^{\theta }$ are indirectly controlled by the asymptotically conservative behavior of JLIM: with sufficiently large effect sizes or sample sizes, the cumulative distribution function of JLIM statistic shifts lower under $H_{2}^{\theta }$ than under *H*_0_ as previously shown analytically [39]. Thus, under the asymptotic condition, JLIM guarantees that the *p*-value estimated under *H*_0_ can be used to control for $H_{1}^{\theta }$ as well. However, under non-asymptotic conditions, in particular in case of underpowered cohorts of limited sample sizes, the power to distinguish $H_{1}^{\theta }$ from $H_{2}^{\theta }$ will be diminished. This reduced specificity is more pronounced when the LD between distinct causative variants for two traits is substantial (See Supplements of [39] for the detail). JLIM assumes that only up to one causative variant is present for each trait in a locus. However, simulations showed that the accuracy of JLIM remained robust in the presence of multiple causative variants in a locus (S12 Fig).

To run JLIM, we used the genetic association statistics of OSA traits calculated over all common SNPs in a 200kb analysis window around the focal SNP (the lead SNP of a clinical trait). We derived these statistics from our discovery cohorts by combining association signals of each cohort using an inverse-variance weighted meta-analysis approach. The association statistics were calculated in individual cohorts by linear regression adjusting for age, sex, BMI and the top three principal components. The principal components were calculated from genome-wide genotype data in each cohort separately. We used mean imputation for missing covariate values. Multi-allelic SNPs and rare variants with minor allele frequencies (MAF) below 0.05 were excluded from the analysis. We only used variants present in all of the discovery cohorts. To reflect the limited resolution for fine-mapping of causative variants in underpowered studies, we relaxed JLIM’s genetic resolution parameter *θ* to 0.5 from the default value of 0.8. All other parameters were set to the default. We ran JLIM only in the discovery cohorts.

JLIM 2.0 requires individual-level genetic data for underpowered traits in order to run permutations. This limits the applicability of our approach to more general cases where only summary-level data are available. The direct permutation on individual-level genetic data, however, enables robust estimation of *p*-values even under the vagaries of imputation noise across cohorts for underpowered traits. For the permutation procedure, we used the same pipeline described above to generate permuted association statistics for JLIM. OSA phenotypes were randomly shuffled in each cohort separately. For each permutation, the association statistics were calculated in the same way including all the covariates. Then, the cohort-level association statistics calculated on permuted data were combined across cohorts by meta-analysis. This permutation procedure was repeated up to 100,000 times, adaptively, to estimate JLIM *p*-values.

We accounted for the multiple testing burden with the False Discovery Rate (FDR), separately for each clinical and OSA trait combination. Specifically, we used the Benjamini-Hochberg procedure to calculate the FDR as follows:

$$FDR_{u,v}(p)=\frac{pN_{u,v}}{\left|\left\{l|{JLIM}_{u,v}(l)<p\right\}\right|}$$

where *u* and *v* indicate a combination of clinical and OSA traits, *JLIM*_*u*,*v*_(*l*) is the JLIM *p*-value of tested locus *l*, and *N*_*u*,*v*_ is the number of loci tested between the trait pairs *u* and *v*. JLIM hits were obtained at the FDR threshold of 0.2. The JLIM/cFDR consensus was defined by the intersection between the JLIM (FDR < 0.2) and cFDR hits defined at the same *p*-value threshold. Specifically, given the JLIM *p*-value cutoff of FDR 0.2, i.e., *p*_0.2_ such that *FDR*_*u*,*v*_(*p*_0.2_) = 0.2, the cFDR hits were defined by the association *p*-value below *p*_0.2_ for an OSA trait. Note that the *p*-value of association to a clinical trait is always ascertained to be less than 5 x 10^−8^ in our analysis. The cFDR association *p*-values were examined at the focal SNP.

### Comparison with eCAVIAR

eCAVIAR (version 2.2) was run in the default setting. The reference LD matrix was set to the LD estimated from the subjects of European ancestry (n = 10,000) randomly subsampled out of our OSA discovery cohort. Colocalization Posterior Probability (CLPP) was calculated for lead SNPs of well-powered clinical traits to identify pleiotropic loci. The CLPP thresholds were calibrated to be equivalent to *p*-value cutoffs using unfiltered null simulation datasets (H_0_), which were not preconditioned on the minimum association *p*-values in loci.

### Replication of OSA associations in independent samples

We validated the OSA associations identified in the discovery cohorts by replicating them in out-of-sample multi-ethnic replication cohorts (S6 Table). There was no sample overlap between our discovery and replication cohorts. We combined the *p*-values of associations across the six replication cohorts by applying an inverse variance-weighted meta-analysis technique. We defined the *p*-value of association < 0.05 as nominal evidence of replication and the *p*-value < 0.05/61 as a more stringent Bonferroni-corrected replication cutoff, given that 61 independent SNPs were uncovered for their pleiotropic associations to OSA in the discovery cohort (Tables 1 and S5).

### Comparison of nominal replication rates with random SNPs

To assess the sensitivity of our pleiotropy analysis, we compared the nominal replication rate of random SNPs with that of candidate pleiotropic variants identified in the discovery cohort. We started with 87,938 SNPs randomly selected across the autosomes, excluding chromosome 6 to avoid the major histocompatibility complex locus. Then, we used Plink to apply LD pruning on the random set of SNPs and obtained a subset of 28,558 independent SNPs. The LD pruning procedure ensured that the *r*^*2*^ between SNPs was less than 0.1 in the distance of 100kb in the LD background of non-Finnish Europeans (n = 404) from the 1000 Genomes Project. For the fair comparison between random and predicted pleiotropic SNPs, we further filtered these random SNPs based on the OSA association *p*-values in 200kb windows. Overall, we kept only 11,910 random independent SNPs that have an AHI-associated SNP (*p* < 0.01) within 100kb distance. On all loci examined for the pleiotropy, we similarly applied the LD pruning procedure on the focal SNPs to obtain a subset of independent SNPs. During the pruning steps, SNPs with more significant JLIM *p*-values were preferentially retained. After the LD pruning, we obtained 995, 1,024, 967 and 961 independent SNPs for AHI, average SpO_2_, minimum SpO_2_ and event duration, respectively; of which 20, 12, 6 and 11 SNPs are predicted to be pleiotropic in the discovery cohort.

### Identifying pleiotropic variants affecting both gene expressions and OSA

We used *cis*-expression quantitative trait loci (eQTLs) from the Gene-Tissue Expression project (GTEx release v8) [63] and BLUEPRINT epigenome project [64], to examine pleiotropy between the variation in gene expression levels and OSA phenotypes. Among the GTEx datasets, we only considered liver (n = 178), spleen (n = 179), skeletal muscle (n = 588) and lung (n = 436) tissues for our analysis, based on the potential relevance of these tissues to OSA and their sample sizes. Again, we used eQTLs calculated only with samples of European ancestry for this analysis. The genome-wide summary statistics of European American eQTLs were obtained from the GTEx Consortium. For the analysis of immune cell eQTLs, we used BLUEPRINT datasets which consisted of genotypes of participants and expression profiles of CD14^+^ monocytes (n = 191), neutrophils (n = 196) and CD4^+^ T cells (n = 167). The RNA transcripts of BLUEPRINT samples were derived from unstimulated primary cells collected from healthy individuals of European ancestry. The genome-wide summary statistics of BLUEPRINT eQTLs were downloaded from the eQTL Catalogue [95].

We start with the 61 index SNPs corresponding to the candidate pleiotropic loci identified by JLIM FDR < 0.2 and cFDR consensus (S5 Table). Using GTEx and BLUEPRINT eQTLs, we scanned for pleiotropy between eQTLs and clinical traits. We considered all protein-coding genes whose transcription start sites (TSS) were less than 1Mb away from the focal SNP of a clinical trait (1,011 unique genes; 5,690 eQTLs). The protein-coding genes were defined by Ensembl annotation (release 104). The genes with eQTL association *p*-value > 0.05 at all SNPs in the locus were excluded due to weak evidence of association to gene expression. Overall, a total of 5,860 clinical traits/eQTLs pairs (1,009 unique genes; 5,023 eQTLs) were tested for the pleiotropy using JLIM in the default setting (Table 2). JLIM version 2.5 was used to test for pleiotropy only using summary-level eQTL data that are publicly available. The *p*-values were calculated by adaptive resampling (up to 10,000 iterations). The null distribution of JLIM statistic was generated by random sampling of phenotypes from a normal distribution, sampling of genotypes from the reference LD panel (1000 Genomes, non-Finnish Europeans), and then linearly regressing the sampled phenotypes on the genotypes. Multiallelic SNPs and rare variants with MAF < 0.05 were excluded from the analysis. The multiple testing burden was accounted for with the False Discovery Rate (FDR) by applying the Benjamini-Hochberg procedure separately to each tissue/cell type and clinical trait combination.

### Using gene expression as clinical traits

In three loci with known genome-wide associations to OSA traits, we tested for pleiotropy between gene expression and OSA traits using *cis*-eQTLs as clinical traits. We examined four GTEx tissues and three BLUEPRINT immune cell types, similarly to the above causal chain analysis. There are a total 57 eQTLs in the three loci satisfying: 1) the eQTL *p*-values < 5 x 10^−8^ for a SNP within the 200kb window centered at the known OSA-associated SNP, and 2) the transcription start sites being within 1 Mb from the OSA-associated SNP. Using these 57 eQTLs as clinical traits, we applied JLIM in the default setting (S7 Table). For the OSA traits, we used the association statistics from our discovery cohorts. The JLIM FDR was calculated using the Benjamini-Hochberg procedure (a total of 57 tests).

### Simulated datasets

To compare the accuracy of JLIM to other methods, we simulated genetic loci with pleiotropic associations under different scenarios with unbalanced sample sizes. For one of two traits, we simulated a well-powered GWAS of a quantitative trait with a sample size of 150,000. For the other trait, we simulated an underpowered GWAS with a much smaller sample size of 10,000. To generate datasets of realistic LD backgrounds, we used real genotypes of 80 randomly picked loci across the genome. Each locus was 200kb in length. Chromosome 6 and the sex chromosomes were excluded from our simulations due to the difference in LD patterns from the rest of genome.

In these loci, we generated 80,000 sets of association statistics under each of the following six scenarios (H_0_, H_1_ and four configurations of H_2_). For the simulations of H_0_, the SNP in the midpoint of the genomic segment was chosen as the causative variant for well-powered traits, but no causal effect was simulated for underpowered traits. For H_1_, the midpoint SNP was taken as the shared causative variant for both well-powered and underpowered traits. For H_2_, the midpoint SNP was selected to be causal for well-powered traits, but a distinct SNP was selected as the causative variant for underpowered traits. The distinct SNP was randomly chosen in the intervals of LD relative to the midpoint SNP, selecting one in each of the following LD ranges: |*r*| < 0.3, 0.3 < |*r*| < 0.6, 0.6 < |*r*| < 0.8 and 0.8 < |*r*| < 0.95.

For each pair of causative variants, we randomly sampled the genetic effect sizes (*β*_1_, *β*_2_), corresponding to well-powered and underpowered traits, respectively, from the following bivariate normal distribution:

$$\left(\beta_{1},\beta_{2})∼N(0,\Sigma \right)$$

$$\Sigma =\left[\begin{array}{cc} \sigma_{1}^{2} & \rho \sigma_{1}\sigma_{2}\\ \rho \sigma_{1}\sigma_{2} & \sigma_{2}^{2} \end{array}\right]$$

where $\sigma_{1}^{2}$ and $\sigma_{2}^{2}$ are per-SNP heritability of two traits, set to 5 x 10^−5^ under the assumptions of the heritability of 0.5, causal fraction of 0.01 and 1,000,000 independent markers across the genome. The parameter *ρ* was used to represent the correlation of causal effect sizes between two traits. For H_1_, we generated simulated data under no correlation (*ρ* = 0) as well as moderate to high correlation (*ρ* = 0.5, 0.7 or 0.9). For H_2_, we assumed *ρ* = 0 since the causative variants are not shared. For H_0_, *β*_2_ was set to 0.

JLIM only requires GWAS summary statistics for the well-powered trait. Therefore, for all SNPs *j* = 1,…,*m* in each locus, we generated summary statistics by sampling the observed association statistics *z* = (*z*_1_,…,*z*_*j*_,…,*z*_*m*_) from the following multivariate normal distribution [52]:

$$z∼N(\sqrt{N_{1}}D\beta ,D)$$

where *N*_1_ is the GWAS sample size (150,000), *m* is the number of markers in the locus, *D* is the *m* x *m* local LD matrix, and *β* is the *m*-dimensional vector of true causal effects (on standardized genotype values) of all SNPs in the locus. Here, *β* was set to *β*_1_ at the causative SNP and to 0 at all other SNPs. The *p*-values of association were calculated from the *z* scores as follows:

$$p_{j}=2\Phi (-|z_{j}|)$$

where *z*_*j*_ is the association statistic at SNP *j* and *Φ* is the standard normal cumulative distribution function.

JLIM 2.0 requires individual-level genotype data as well as association statistics for the second trait. Therefore, for the underpowered trait, we generated a discovery cohort with simulated phenotypes for all individuals; then, we calculated the association statistics by linear regression between genotypes and phenotypes, instead of sampling the summary statistics from a multivariate normal distribution. The genotype data of 10,000 individuals were obtained by subsampling from six cohorts of European Ancestry (MESA, ARIC, MrOS, CHS, CFS, FHS and WASHS). The phenotype value *y*_*i*_ of each individual *i* was generated by random sampling from the following standard normal distribution:

$$y_{i}∼N(x_{i}\beta_{2},1)$$

where *x*_*i*_ is the genotype of the causative variant in the individual *i* (standardized to the mean of 0 and the variance of 1). The association statistics and *p*-values of association were obtained by regressing the phenotypes *y*_*i*_ on the genotypes of each SNP in the locus. We excluded multiallelic sites and variants with MAF < 0.05 from the analysis.

The above process generated up to 480,000 sets of association data under scenarios of H_0_, H_1_ and H_2_ in the genetic background of 80 loci (i.e., 80 x 6 x 1,000). Because of sampling noise, in a minority of simulations, association data of some loci failed to pass the genome-wide significance threshold for well-powered traits. We rejected such simulation runs to mimic our data analysis, where we only included loci in which the clinical trait association *p*-value was genome-wide significant. Similarly, we excluded instances where there exists no SNP with the *p*-value of association < 0.01 for underpowered traits. In total, we retained 25,958, 31,858, 28,602, 29,131, 27,025 and 25,115 sets of association data for H_0_, H_1_, and H_2_ of distinct causative variants in low to high LD, respectively.

Next, we estimated the distribution of LD between random pairs of SNPs in the 80 simulated loci and titrated the causative variants of H_2_ simulations to follow this distribution. Specifically, we subsampled H_2_ datasets to fit to the following composition: 72% for |*r*| < 0.3, 19% for 0.3 < |*r*| < 0.6, 5% for 0.6 < |*r*| < 0.8 and 4% for 0.8 < |*r*| < 0.95 (S2C Fig). After this, we generated GWAS datasets of *T* loci by random subsampling of H_0_, H_1_ and H_2_. *T* represents the total number of GWAS loci available from well-powered traits and varied from 500 to 5,000. The proportion of H_0_ out of *T* loci varied from 0 to 30%. The relative ratios of loci simulating H_1_ and H_2_ were set to 1:19, 1:9, 1:4 or 1:0.

To assess the power to replicate the pleiotropic associations in independent validation cohorts, we calculated the expected association statistic $z_{j}^{v}$ for the underpowered trait at the focal SNP *j*:

$$E[z_{j}^{v}]=\sqrt{N_{v}}\beta_{2}r_{jj\prime }$$

where the focal SNP *j* was defined by the lowest *p*-value of association to the well-powered trait, *N*_*v*_ is the sample size of validation cohort for the underpowered trait, ranging from 10,000 to 35,000, *β*_2_ is the effect size for the underpowered trait at its causative SNP *j*′, and *r*_*jj*′_ is the LD between two SNPs *j* and *j*′. For the LD backgrounds of validation cohorts, we used CEU and YRI from the 1000 Genomes Project to represent ancestry-matched and trans-ethnic validation cohorts, respectively. The expected number of replicated signals after Bonferroni correction was calculated by estimating:

$$E\left[2\Phi \left(-|E[z_{j}^{v}]|\right)<\frac{0.05}{M}\right]$$

where *M* is the number of pleiotropic associations found in each subsampled GWAS dataset of *T* loci.

Last, we also evaluated the accuracy of our method under the presence of multiple causative variants that were shared between two traits in H_1_. In addition to the midpoint SNP, we randomly picked another SNP in the locus as a second shared causative variant. The effect sizes of the two causative variants for well-powered and underpowered traits were sampled from the same bivariate normal distribution. We generated GWAS datasets for the well-powered and underpowered traits in a similar manner as the simulations of single causative variants. The correlation of effect sizes between well-powered and underpowered traits (*ρ*) was set to 0.7 for H_1_. The proportion of H_0_ was assumed to be 30% of examined GWAS loci (2,500). The relative proportion of H_1_ and H_2_ was set to 1:19. The proportion of loci with two causative variants was set to 1/4 of all H_1_ as expected under Poisson distribution with the causal fraction of 0.01.

### Simulated datasets for the meta-analysis of more than two traits

GWAS data were simulated for ten well-powered and one underpowered traits under H_0_, H_1_ and H_2_ (S15A Fig). One of the well-powered traits was ascertained to have a genome-wide significant association (the main well-powered trait). Here, we simulated association statistics only for the causative SNP of the main well-powered trait rather than simulating for entire SNPs in the locus. For simplicity of simulation, we assumed that the lead SNP of the main well-powered trait is in tight LD with its causative variant. The rest of well-powered traits have the same or distinct causative variants from the main well-powered trait. The number of additional well-powered traits simulating the same causative variants was randomly decided by sampling from a binomial distribution $Binom\left(n=9,p=\frac{1}{20}\right)$, and the rest were assumed to have distinct causative variants. For underpowered traits, the causative variant was assumed to be absent, same or distinct from the main well-powered trait depending on whether the locus was simulated under H_0_, H_1_ or H_2_, respectively. Effect sizes of all traits sharing the same causative variant were sampled together from a multivariate normal distribution with the correlation of effect sizes between traits set to 0.7. Effect sizes of traits simulating distinct causative variants were sampled independently and then multiplied by a random variable representing the LD between causative and tested SNPs. The association statistics were generated with the sample sizes of n = 150,000 for well-powered traits and n = 10,000 for underpowered traits. In total, 3,751, 613 and 6,229 sets of simulated association statistics were generated under H_0_, H_1_ and H_2_, respectively. From these simulated H_0_, H_1_ and H_2_ datasets, GWAS datasets of 2,500 loci were generated by random subsampling at the proportions of 30%, 3.5% and 66.5%, respectively (H_1_:H_2_ ratio of 1:19). We evaluated the power to replicate the candidate pleiotropic loci using the subsampled GWAS datasets (1,000 iterations). The power to replicate after Bonferroni correction was estimated in the same way as other simulations. The validation cohort were assumed to be of the same genetic ancestry as the discovery cohort, and the sample sizes varied from n = 10,000 to 35,000. For pairwise analyses, we used only the main well-powered trait and underpowered trait. On the other hand, for full multi-trait analyses, we used the entire data including all ten well-powered traits and one underpowered trait.

### Meta-analysis methods

We benchmarked the power to replicate pleiotropic associations in underpowered cohorts by applying three meta-analysis methods–MetABF, CPBayes and iGWAS–on simulated datasets. We ran MetABF in the subset-exhaustive mode to scan all possible subsets of pleiotropic traits. The parameter for the correlation of effect sizes was set to the known value of simulation (*ρ* = 0.7). MetABF was run three times with the scale parameter of causal effect sizes set to 0.1, 0.2 and 0.4, and the Approximate Bayes Factors (ABF) were averaged over the three runs as recommended by the authors. MetABF reports the ABF relative to H_0_, thus we calculated the ABF of pleiotropy to the underpowered trait relative to that of no pleiotropy to the underpowered trait by summing ABFs over subsets. In contrast, we ran CPBayes in the fully automatic setting. All parameters of the pleiotropy were directly learned from the data. We used the PPAj (Posterior Probability of Association) of the underpowered trait to identify pleiotropic associations. In comparison to the two Bayesian methods, iGWAS is a non-parametric meta-analysis test. We ran iGWAS in the default setting. For all three methods, candidate pleiotropic associations were identified in the discovery cohort at the same *p*-value cutoff of 0.01. For the Bayesian methods, we applied the equivalent ABF and PPAj thresholds by calibrating them to have the false positive rate of 0.01 in our null simulation (H_0_).

## Supporting information

S1 Acknowledgments Full list of acknowledgments for funding for data used in this study. (DOCX)

S1 Fig Schematic of JLIM analysis. Examples of trait pairs simulated under the null (no causal effect for an underpowered trait, H_0_), shared effect between well-powered and underpowered traits (H_1_), and distinct effects between two compared traits (H_2_). H_1_ and H_2_ are competing alternative hypotheses. The simulated true causal variants are indicated by red arrows.
(TIF)

S2 Fig Sensitivity for H_1_ and specificity to distinguish H_1_ from H_2_. **(A)** Sensitivity to detect H_1_ at the same false positive rate for H_0_. The same *p*-value cutoff was used for JLIM and cFDR. For eCAVIAR, a posterior cutoff was calibrated to match the *p*-value cutoff using unfiltered H_0_ simulation data. **(B)** The rate of misclassifying H_2_ as H_1_. H_2_ loci include all loci simulating distinct causative variants in LD between 0 to 0.95. Again, the cutoffs of cFDR, eCAVIAR and JLIM were calibrated to the same specificity using H_0_. **(C)** The distribution of LD for randomly selected pairs of SNPs. This distribution has been drawn from the LD patterns between random pairs of SNPs within 80 random loci (200kb each) in the population of European ancestry. This distribution was used to simulate the LD between distinct causative variants in H_2_. **(D,E,F)** The rate of misclassifying H_2_ as H_1_, broken down by the LD between simulated distinct causative variants for two traits. The cutoff of JLIM *p*-values was set to **(D)** 0.05, **(E)** 0.01 or **(F)** 0.005. The dashed horizontal line indicates the JLIM *p*-value cutoff. θ represents the genetic resolution parameter for JLIM, set to 0.5 in this study. JLIM does not claim to distinguish H_2_ beyond the specified genetic resolution limit (*r*^*2*^ > θ; light grey bars).
(TIF)

S3 Fig Winner’s curse. The true and observed genetic effect sizes for underpowered traits are shown to highlight the winner’s curse in a discovery cohort. We show data from 10,000 loci each simulated under **(A)** H_1_ and **(B)** H_2_. In all panels, each dot represents the genetic effects measured at the index SNPs. The index SNPs are defined as the lead SNPs of association to well-powered clinical traits. Occasionally, the index SNPs deviate from the causative SNPs due to sampling noise, and when this happens, we calculated the true effect size at the index SNP by multiplying the true effect of the causative SNP by the LD between the index and causative SNPs. The loci detected at JLIM *p* < 0.01 are indicated by cyan dots, and their density distribution are shown in contours. The loci found by cFDR (association *p* < 5 x 10^−8^ for well-powered trait and < 0.01 for underpowered trait) are represented by dots above the black horizontal line.
(TIF)

S4 Fig The number of loci that were identified in simulated discovery and validation cohorts, broken down by the configuration of causative variants. **(A)** The number of loci identified in a discovery cohort by pleiotropy analysis. **(B-F)** The number of loci replicated in a validation cohort, subdivided by the configuration of causative variants: **(B)** H_1_, H_2_ with the LD between causative variants to be in the ranges of **(C)** |*r*| < 0.3, **(D)** 0.3 < |*r*| < 0.6, **(E)** 0.6 < |*r*| < 0.8 and **(F)** 0.8 < |*r*| < 0.95. In all panels, simulation was conducted under the following parameters: A total of 2,500 association peaks from well-powered GWAS studies (n = 150,000) were tested for pleiotropy in simulated discovery cohorts (n = 10,000), and then the candidate pleiotropic loci were tested for replication in simulated validation cohorts of the same genetic ancestry (n = 10,000–35,000). The candidate loci were identified by conditional false discovery rate (cFDR), eCAVIAR, Joint Likelihood Mapping (JLIM), or the intersection of eCAVIAR or JLIM and cFDR, all at the *p*-value cutoff of 0.01 (or equivalent posterior cutoff). The 2,500 GWAS peaks consist of the loci simulating no causal effect for underpowered traits (H_0_) and those simulating the same causal effect between two traits (H_1_) or distinct causal effects (H_2_). The proportion of H_0_ was set to 30%, and the remaining 70% of loci were split to H_1_ and H_2_ at the ratio of 1:19. The effect sizes of causative variants are correlated (*ρ* = 0.7) under H_1_ but uncorrelated under H_2_. Bonferroni correction was applied on replication tests. The shaded area denotes the 95% CIs.
(TIF)

S5 Fig The projected number of replicated loci in simulations, varying the relative ratio between H_1_ and H_2_. A total of 2,500 association peaks from well-powered GWAS studies (n = 150,000) were tested for pleiotropy in a discovery cohort (n = 10,000), and then the candidate pleiotropic loci were tested for replication in an independent validation cohort of the same genetic ancestry (n = 10,000–35,000). The candidate loci were identified by conditional false discovery rate (cFDR), Joint Likelihood Mapping (JLIM), or the intersection of both, all at the *p*-value cutoff of 0.01. The 2,500 GWAS peaks consist of the loci simulating no causal effect for underpowered traits (H_0_) and those simulating the same causal effect between two traits (H_1_) or distinct causal effects (H_2_). The proportion of H_0_ was set to 30%, and the remaining 70% of loci were split to H_1_ and H_2_ at the ratio of **(A)** 1:19, **(B)** 1:9, **(C)** 1:4, and **(D)** 1:0. The effect sizes of causative variants are correlated (*ρ* = 0.7) under H_1_ but uncorrelated under H_2_. Bonferroni correction was applied on replication tests. The shaded area denotes the 95% CIs.
(TIF)

S6 Fig The projected number of replicated loci in simulations, varying the proportion of H_0_ loci. A total of 2,500 association peaks from well-powered GWAS studies (n = 150,000) were tested for pleiotropy in a discovery cohort (n = 10,000), and then the candidate pleiotropic loci were tested for replication in an independent validation cohort of the same genetic ancestry (n = 10,000–35,000). The candidate loci were identified by conditional false discovery rate (cFDR), Joint Likelihood Mapping (JLIM), or the intersection of both, all at the *p*-value cutoff of 0.01. The 2,500 GWAS peaks consist of the loci simulating no causal effect for underpowered traits (H_0_) and those simulating the same causal effect between two traits (H_1_) or distinct causal effects (H_2_). The proportion of H_0_ was varied to **(A)** 0%, **(B)** 10%, **(C)** 20% and **(D)** 30%, and the remaining loci were split to H_1_ and H_2_ at the ratio of 1:19. The effect sizes of causative variants are correlated (ρ = 0.7) under H_1_ but uncorrelated under H_2_. Bonferroni correction was applied on replication tests. The shaded area denotes the 95% CIs.
(TIF)

S7 Fig The projected number of replicated loci in simulations where the cFDR threshold was tightened to identify the same number of candidate loci as the JLIM/cFDR consensus method in a discovery cohort. A total of 2,500 association peaks from well-powered GWAS studies (n = 150,000) were tested for pleiotropy in a discovery cohort (n = 10,000), and then the candidate pleiotropic loci were tested for replication in an independent validation cohort of the same genetic ancestry (n = 10,000–35,000). The candidate loci were identified by conditional false discovery rate (cFDR) or by the JLIM/cFDR consensus method. **(A)** The consensus method (red line) selected 12.9 candidate pleiotropic loci by taking the intersection between JLIM *p* < 0.01 and cFDR (association *p* < 0.01 for an underpowered trait). For the comparison, we tightened cFDR threshold to underpowered trait assoc *p* < 0.0012 so to identify the same number of candidate loci in a discovery cohort (black line). **(B)** Similarly, the consensus method (red line) found 7.5 candidate loci by taking the intersection between JLIM *p* < 0.005 and cFDR (assoc *p* < 0.005 for underpowered trait). The cFDR threshold was tightened to underpowered trait assoc *p* < 0.00060 for the same number of candidates (black line). In both panels, the 2,500 GWAS peaks consist of the loci simulating no causal effect for underpowered traits (H_0_) and those simulating the same causal effect between two traits (H_1_) or distinct causal effects (H_2_). The proportion of H_0_ was set to 30%, and the remaining 70% of loci were split to H_1_ and H_2_ at the ratio of 1:19. The effect sizes of causative variants are correlated (ρ = 0.7) under H_1_ but uncorrelated under H_2_. Bonferroni correction was applied on replication tests. The shaded area denotes the 95% CIs.
(TIF)

S8 Fig The projected number of replicated loci in simulations where the cFDR threshold was calibrated to have the same false positive rate for H_0_. A total of 2,500 association peaks from well-powered GWAS studies (n = 150,000) were tested for pleiotropy in a discovery cohort (n = 10,000), and then the candidate pleiotropic loci were tested for replication in an independent validation cohort of the same genetic ancestry (n = 10,000–35,000). The candidate loci were identified by conditional false discovery rate (cFDR) or by the JLIM/cFDR consensus method. **(A)** The consensus method (red line), by taking the intersection between JLIM *p* < 0.01 and cFDR *p* < 0.01, showed the empirical false positive rate of 0.0038 in simulated H_0_ dataset. To match this false positive rate, we tightened cFDR threshold to *p* < 0.0038 (black line). **(B)** Similarly, the consensus method (red line) showed the empirical false positive rate of 0.0018 in H_0_ when the intersection was taken at JLIM and cFDR *p* < 0.005. To match the false positive rate, the cFDR threshold was tightened to *p* < 0.0018 (black line). The cFDR *p*-value refers to the *p*-value of association to an underpowered trait. In both panels, the 2,500 GWAS peaks consist of the loci simulating no causal effect for underpowered traits (H_0_) and those simulating the same causal effect between two traits (H_1_) or distinct causal effects (H_2_). The proportion of H_0_ was set to 30%, and the remaining 70% of loci were split to H_1_ and H_2_ at the ratio of 1:19. The effect sizes of causative variants are correlated (ρ = 0.7) under H_1_ but uncorrelated under H_2_. Bonferroni correction was applied on replication tests. The shaded area denotes the 95% CIs.
(TIF)

S9 Fig The projected number of replicated loci in simulations where the number of association peaks available from well-powered traits varied. A total of **(A)** 500, **(B)** 1,000, **(C)** 2,500 and **(D)** 5,000 association peaks from well-powered GWAS studies (n = 150,000) were tested for pleiotropy in a discovery cohort (n = 10,000), and then the candidate pleiotropic loci were tested for replication in an independent validation cohort of the same genetic ancestry (n = 10,000–35,000). The candidate loci were identified by conditional false discovery rate (cFDR) or by the JLIM/cFDR consensus method. The consensus method (red line), by taking the intersection between JLIM *p* < 0.01 and cFDR *p* < 0.01, showed the empirical false positive rate of 0.0038 in simulated H_0_ dataset. To match this false positive rate, we tightened cFDR threshold to *p* < 0.0038 (black line). The cFDR *p*-value refers to the *p*-value of association to an underpowered trait. The GWAS peaks consist of the loci simulating no causal effect for underpowered traits (H_0_) and those simulating the same causal effect between two traits (H_1_) or distinct causal effects (H_2_). The proportion of H_0_ was set to 30%, and the remaining 70% of loci were split to H_1_ and H_2_ at the ratio of 1:19. The effect sizes of causative variants are correlated (ρ = 0.7) under H_1_ but uncorrelated under H_2_. Bonferroni correction was applied on replication tests. The shaded area denotes the 95% CIs.
(TIF)

S10 Fig The projected number of replicated loci in simulations where the correlation of effect sizes for H_1_ varied. A total of 2,500 association peaks from well-powered GWAS studies (n = 150,000) were tested for pleiotropy in a discovery cohort (n = 10,000), and then the candidate pleiotropic loci were tested for replication in an independent validation cohort of the same genetic ancestry (n = 10,000–35,000). The candidate loci were identified by conditional false discovery rate (cFDR) or by the JLIM/cFDR consensus method. The consensus method (red line), by taking the intersection between JLIM *p* < 0.01 and cFDR *p* < 0.01, showed the empirical false positive rate of 0.0038 in simulated H_0_ dataset. To match this false positive rate, we tightened cFDR threshold to *p* < 0.0038 (black line). The cFDR *p*-value refers to the *p*-value of association to an underpowered trait. The 2,500 GWAS peaks consist of the loci simulating no causal effect for underpowered traits (H_0_) and those simulating the same causal effect between two traits (H_1_) or distinct causal effects (H_2_). The proportion of H_0_ was set to 30%, and the remaining 70% of loci were split to H_1_ and H_2_ at the ratio of 1:19. The effect sizes of causative variants are correlated with **(A)** ρ = 0.0, **(B)** ρ = 0.5, **(C)** ρ = 0.7 and **(D)** ρ = 0.9 under H_1_ but uncorrelated under H_2_. In all panels, Bonferroni correction was applied on replication tests. The shaded area denotes the 95% CIs.
(TIF)

S11 Fig The projected number of replicated loci in simulations of trans-ethnic replication. A total of 2,500 association peaks from well-powered GWAS studies (n = 150,000) were tested for pleiotropy in a discovery cohort (n = 10,000), and then the candidate pleiotropic loci were tested for replication in an independent validation cohort of **(A)** the same (CEU) and **(B)** different (YRI) genetic ancestry (n = 10,000–35,000). The LD patterns of genetic ancestry were obtained from the 1000 Genomes Project data. In both panels, the candidate loci were identified by conditional false discovery rate (cFDR) or by the JLIM/cFDR consensus method. The consensus method (red line), by taking the intersection between JLIM *p* < 0.01 and cFDR *p* < 0.01, showed the empirical false positive rate of 0.0038 in simulated H_0_ dataset. To match this false positive rate, we tightened cFDR threshold to *p* < 0.0038 (black line). The cFDR *p*-value refers to the *p*-value of association to an underpowered trait. The 2,500 GWAS peaks consist of the loci simulating no causal effect for underpowered traits (H_0_) and those simulating the same causal effect between two traits (H_1_) or distinct causal effects (H_2_). The proportion of H_0_ was set to 30%, and the remaining 70% of loci were split to H_1_ and H_2_ at the ratio of 1:19. The effect sizes of causative variants are correlated with ρ = 0.7 under H_1_ but uncorrelated under H_2_. Bonferroni correction was applied on replication tests. The shaded area denotes the 95% CIs.
(TIF)

S12 Fig The projected number of replicated loci in simulations of multiple causative variants for H_1_. A total of 2,500 association peaks from well-powered GWAS studies (n = 150,000) were tested for pleiotropy in a discovery cohort (n = 10,000), and then the candidate pleiotropic loci were tested for replication in an independent validation cohort of the same genetic ancestry (n = 10,000–35,000). The candidate loci were identified by conditional false discovery rate (cFDR) or by the JLIM/cFDR consensus method. The consensus method (red line), by taking the intersection between JLIM *p* < 0.01 and cFDR *p* < 0.01, showed the empirical false positive rate of 0.0038 in simulated H_0_ dataset. To match this false positive rate, we tightened cFDR threshold to *p* < 0.0038 (black line). The cFDR *p*-value refers to the *p*-value of association to an underpowered trait. The 2,500 GWAS peaks consist of the loci simulating no causal effect for underpowered traits (H_0_) and those simulating the same causal effect between two traits (H_1_) or distinct causal effects (H_2_). The proportion of H_0_ was set to 30%, and the remaining 70% of loci were split to H_1_ and H_2_ at the ratio of 1:19. In Panel **(A)**, only one causative variant was simulated for H_1_, whereas in Panel **(B)**, up to two causative variants were simulated for H_1_. The proportion of loci with two causative variants was set to 1/4 of all H_1_ as expected under Poisson distribution with the causal fraction of 0.01. The effect sizes of causative variants are correlated with ρ = 0.7 under H_1_ but uncorrelated under H_2_. Bonferroni correction was applied on replication tests. The shaded area denotes the 95% CIs.
(TIF)

S13 Fig Comparison with Bayesian meta-analysis methods with a validation cohort from the same ancestry. A total of 2,500 association peaks from well-powered GWAS studies (n = 150,000) were tested for pleiotropy in a discovery cohort (n = 10,000), and then the candidate pleiotropic loci were tested for replication in an independent validation cohort of the same genetic ancestry (n = 10,000–35,000). The candidate loci were identified by the JLIM/cFDR consensus method, MetABF and CPBayes. The consensus method (red line), by taking the intersection between JLIM *p* < 0.01 and cFDR *p* < 0.01, showed the empirical false positive rate of 0.0038 in simulated H_0_ dataset. Bayesian posterior thresholds for MetABF (green) and CPBayes (purple) calibrated using H_0_ loci to match the false positive rate of the consensus method. The 2,500 GWAS peaks consist of the loci simulating no causal effect for underpowered traits (H_0_) and those simulating the same causal effect between two traits (H_1_) or distinct causal effects (H_2_). The proportion of H_0_ varied to **(A)** 0%, **(B)** 10%, **(C)** 20% and **(D)** 30%, and the remaining loci were split to H_1_ and H_2_ at the ratio of 1:19. In all panels, the effect sizes of causative variants are correlated with ρ = 0.7 under H_1_ but uncorrelated under H_2_. Bonferroni correction was applied on replication tests. The shaded area denotes the 95% CIs. In **(E)**, the fraction of H_1_ among replicated loci is compared among three methods when the proportion of H_0_ is 30% (similar for different proportions of H_0_).
(TIF)

S14 Fig Comparison with Bayesian meta-analysis methods with a validation cohort from the different ancestry (YRI). A total of 2,500 association peaks from well-powered GWAS studies (n = 150,000) were tested for pleiotropy in a discovery cohort (n = 10,000), and then the candidate pleiotropic loci were tested for replication in an independent validation cohort of the different genetic ancestry (YRI; n = 10,000–35,000). The candidate loci were identified by the JLIM/cFDR consensus method, MetABF and CPBayes. The consensus method (red line), by taking the intersection between JLIM *p* < 0.01 and cFDR *p* < 0.01, showed the empirical false positive rate of 0.0038 in simulated H_0_ dataset. Bayesian posterior thresholds for MetABF (green) and CPBayes (purple) calibrated using H_0_ loci to match the false positive rate of the consensus method. The 2,500 GWAS peaks consist of the loci simulating no causal effect for underpowered traits (H_0_) and those simulating the same causal effect between two traits (H_1_) or distinct causal effects (H_2_). The proportion of H_0_ varied to **(A)** 0%, **(B)** 10%, **(C)** 20% and **(D)** 30%, and the remaining loci were split to H_1_ and H_2_ at the ratio of 1:19. In all panels, the effect sizes of causative variants are correlated with ρ = 0.7 under H_1_ but uncorrelated under H_2_. Bonferroni correction was applied on replication tests. The shaded area denotes the 95% CIs. In **(E)**, the fraction of H_1_ among replicated loci is compared among three methods when the proportion of H_0_ is 30% (similar for different proportions of H_0_).
(TIF)

S15 Fig Simulated power of multi-trait meta-analysis compared to pairwise analysis. Ten well-powered traits and one underpowered trait were simulated under H_0_, H_1_ and H_2_. One of the well-powered traits is ascertained to have a genome-wide significant association peak, and the rest of well-powered traits were randomly decided to have the same or distinct causative variants by sampling from a binomial distribution Binom(n = 9, p = 1/20). The underpowered trait harbors no causative variant, the same causative variant or distinct causative variant depending on whether it is simulating H_0_, H_1_ or H_2_. Effect sizes of all traits sharing the same causative variant were sampled together from a multivariate normal distribution with the correlation parameter of 0.7. Effect sizes of traits simulating distinct causative variants were sampled independently. GWAS association statistics at the focal SNP were generated with the sample sizes of n = 150,000 for the well-powered traits and n = 10,000 for the underpowered trait. The validation cohort of the same ancestry was simulated with the sample sizes of n = 10,000 to 35,000. From these simulated H_0_, H_1_ and H_2_ loci, a total of 2,500 GWAS loci were randomly selected at the proportions of 30%, 3.5% and 66.5%, respectively (H_1_:H_2_ ratio of 1:19). **(B)** We applied iGWAS, CPBayes and MetABF on these data. Dashed lines indicate pairwise analyses for which only one genome-wide significant well-powered trait was compared with an underpowered trait. Solid lines indicate multi-trait analyses using the full set of ten well-powered traits and one underpowered trait. Candidate pleiotropic loci were selected for replication analysis at the cutoffs of *p*-value of 0.01 (iGWAS), or equivalent posterior probability thresholds calibrated in the H_0_ dataset (CPBayes and MetABF). Bonferroni correction was applied on replication tests in the validation cohort.
(TIF)

S16 Fig Pleiotropic loci identified by the intersection of JLIM and cFDR are enriched with SNPs that nominally replicate out of sample. The plot shows the fractions of randomly selected and putative pleiotropic loci with OSA associations that are nominally replicated in the meta-analyzed independent validation cohort (S6 Table).
(TIF)

S1 Table Sample sizes of discovery cohorts used for the pleiotropy analysis. (XLSX)

S2 Table Clinical traits used for the pleiotropy analysis. (XLSX)

S3 Table Mendelian randomization analysis *p*-values testing effect of exposure on outcome. (XLSX)

S4 Table Overall number of total tests, identified pleiotropic loci, and nominally replicated signals. (XLSX)

S5 Table List of identified pleiotropic loci and their replication *p*-values for the corresponding OSA traits (JLIM FDR < 0.2). (XLSX)

S6 Table Sample sizes of validation cohorts used for the replication study. (XLSX)

S7 Table Full results of pleiotropy tests between eQTLs and OSA traits in three known OSA GWAS loci at the eQTL *p*-value < 5e-8. (XLSX)
