# Supplementary material for: Leveraging pleiotropy to discover and interpret GWAS results for sleep-associated traits
Source: PLoS Genet. 2022 Dec 27;18(12):e1010557. doi: 10.1371/journal.pgen.1010557 (PMC9829185; doi:10.1371/journal.pgen.1010557)
Supplement: S1 Acknowledgments — (DOCX) [file pgen.1010557.s001.docx]

**S1 Acknowledgments**

The Atherosclerosis Risk in Communities (ARIC) study has been funded in whole or in part with Federal funds from the National Heart, Lung, and Blood Institute, National Institutes of Health, Department of Health and Human Services (contract numbers HHSN268201700001I, HHSN268201700002I, HHSN268201700003I, HHSN268201700004I and HHSN268201700005I), R01HL087641, R01HL059367 and R01HL086694; National Human Genome Research Institute contract U01HG004402; and National Institutes of Health contract HHSN268200625226C. The authors thank the staff and participants of the ARIC study for their important contributions. Infrastructure was partly supported by Grant Number UL1RR025005, a component of the National Institutes of Health and NIH Roadmap for Medical Research.

MESA and the MESA SHARe projects are conducted and supported by the National Heart, Lung, and Blood Institute (NHLBI) in collaboration with MESA investigators. Support for MESA is provided by contracts 75N92020D00001, HHSN268201500003I, N01-HC-95159, 75N92020D00005, N01-HC-95160, 75N92020D00002, N01-HC-95161, 75N92020D00003, N01-HC-95162, 75N92020D00006, N01-HC-95163, 75N92020D00004, N01-HC-95164, 75N92020D00007, N01-HC-95165, N01-HC-95166, N01-HC-95167, N01-HC-95168, N01-HC-95169, UL1-TR-000040, UL1-TR-001079, and UL1-TR-001420, UL1TR001881, DK063491, and R01HL105756. Funding for SHARe genotyping was provided by NHLBI Contract N02-HL-64278. Genotyping was performed at Affymetrix (Santa Clara, California, USA) and the Broad Institute of Harvard and MIT (Boston, Massachusetts, USA) using the Affymetrix Genome-Wide Human SNP Array 6.0. The authors thank the other investigators, the staff, and the participants of the MESA study for their valuable contributions. A full list of participating MESA investigators and institutes can be found at <http://www.mesa-nhlbi.org>.

The Osteoporotic Fractures in Men (MrOS) Study is supported by NIH funding. The following institutes provide support: the National Institute on Aging (NIA), the National Institute of Arthritis and Musculoskeletal and Skin Diseases (NIAMS), NCATS, and NIH Roadmap for Medical Research under the following grant numbers: U01 AG027810, U01 AG042124, U01 AG042139, U01 AG042140, U01 AG042143, U01 AG042145, U01 AG042168, U01 AR066160, and UL1 TR000128. The NHLBI provides funding for the MrOS Sleep ancillary study "Outcomes of Sleep Disorders in Older Men" under the following grant numbers: R01 HL071194, R01 HL070848, R01 HL070847, R01 HL070842, R01 HL070841, R01 HL070837, R01 HL070838, and R01 HL070839. The NIAMS provides funding for the MrOS ancillary study ‘Replication of candidate gene associations and bone strength phenotype in MrOS’ under the grant number R01 AR051124. The NIAMS provides funding for the MrOS ancillary study ‘GWAS in MrOS and SOF’ under the grant number RC2 AR058973. The investigators website can be found at <https://mrosonline.ucsf.edu/>.

This CHS research was supported by NHLBI contracts HHSN268201200036C, HHSN268200800007C, HHSN268201800001C, N01HC55222, N01HC85079, N01HC85080, N01HC85081, N01HC85082, N01HC85083, N01HC85086; and NHLBI grants U01HL080295, R01HL087652, R01HL105756, R01HL103612, R01HL120393, and U01HL130114 with additional contribution from the National Institute of Neurological Disorders and Stroke (NINDS). Additional support was provided through R01AG023629 from the National Institute on Aging (NIA). A full list of principal CHS investigators and institutions can be found at CHS-NHLBI.org. The provision of genotyping data was supported in part by the National Center for Advancing Translational Sciences, CTSI grant UL1TR001881, and the National Institute of Diabetes and Digestive and Kidney Disease Diabetes Research Center (DRC) grant DK063491 to the Southern California Diabetes Endocrinology Research Center. The content is solely the responsibility of the authors and does not necessarily represent the official views of the National Institutes of Health.

Funding for the Western Australian Sleep Health Study was obtained from the Sir Charles Gairdner and Hollywood Private Hospital Research Foundations, the Western Australian Sleep Disorders Research Institute, and the Centre for Genetic Epidemiology and Biostatistics at the University of Western Australia. Funding for the GWAS genotyping obtained from the Ontario Institute for Cancer Research and a McLaughlin Centre Accelerator Grant from the University of Toronto.

The Hispanic Community Health Study/Study of Latinos was carried out as a collaborative study supported by contracts from the NHLBI to the University of North Carolina (HHSN268201300001I / N01-HC65233), University of Miami (HHSN268201300004I / N01-HC65234), Albert Einstein College of Medicine (HHSN268201300002I / N01-HC65235), University of Illinois at Chicago (HHSN268201300003I), Northwestern University (N01-HC65236), and San Diego State University (HHSN268201300005I / N01-HC65237). The following Institutes/Centers/Offices contribute to the HCHS/SOL through a transfer of funds to the NHLBI: National Institute on Minority Health and Health Disparities, National Institute on Deafness and Other Communication Disorders, National Institute of Dental and Craniofacial Research, National Institute of Diabetes and Digestive and Kidney Diseases, National Institute of Neurological Disorders and Stroke, NIH Institution-Office of Dietary Supplements. The Genetic Analysis Center at Washington University was supported by NHLBI and NIDCR contracts (HHSN268201300005C AM03 and MOD03). The views expressed in this manuscript are those of the authors and do not necessarily represent the views of the National Heart, Lung, and Blood Institute; the National Institutes of Health; or the U.S. Department of Health and Human Services. The authors thank the staff and participants of HCHS/SOL for their important contributions. Investigator’s website - <http://www.cscc.unc.edu/hchs/>.

The Starr County Health Studies is supported in part by grants R01 DK073541, U01 DK085501, R01 AI085014, and R01 HL102830 from the National Institutes of Health, and funds from the University of Texas Health Science Center at Houston. We thank the field staff in Starr County for their careful collection of these data and are especially grateful to the participants who so graciously cooperated and gave of their time.

The Cleveland Family Study has been supported by National Institutes of Health grants [5-R01-HL046380-15, 5-KL2-RR024990-05, R35 HL135818, and HL113338].

The Framingham Heart Study (FHS) has been supported by contracts N01-HC-25195 and HHSN268201500001I and grant R01 HL092577. The Framingham Heart Study thanks the study participants and the multitude of investigators who over its 70 year history continue to contribute so much to further our knowledge of heart, lung, blood and sleep disorders and associated traits.

This study makes use of data generated by the BLUEPRINT Consortium. A full list of the investigators who contributed to the generation of the data is available from www.blueprint-epigenome.eu. Funding for the project was provided by the European Union's Seventh Framework Programme (FP7/2007-2013) under grant agreement no 282510 BLUEPRINT.

The Genotype-Tissue Expression (GTEx) Project was supported by the Common Fund of the Office of the Director of the National Institutes of Health, and by NCI, NHGRI, NHLBI, NIDA, NIMH, and NINDS.
