# Supplementary figures and images for: Leveraging pleiotropy to discover and interpret GWAS results for sleep-associated traits
Source: PLoS Genet. 2022 Dec 27;18(12):e1010557. doi: 10.1371/journal.pgen.1010557 (PMC9829185; doi:10.1371/journal.pgen.1010557)

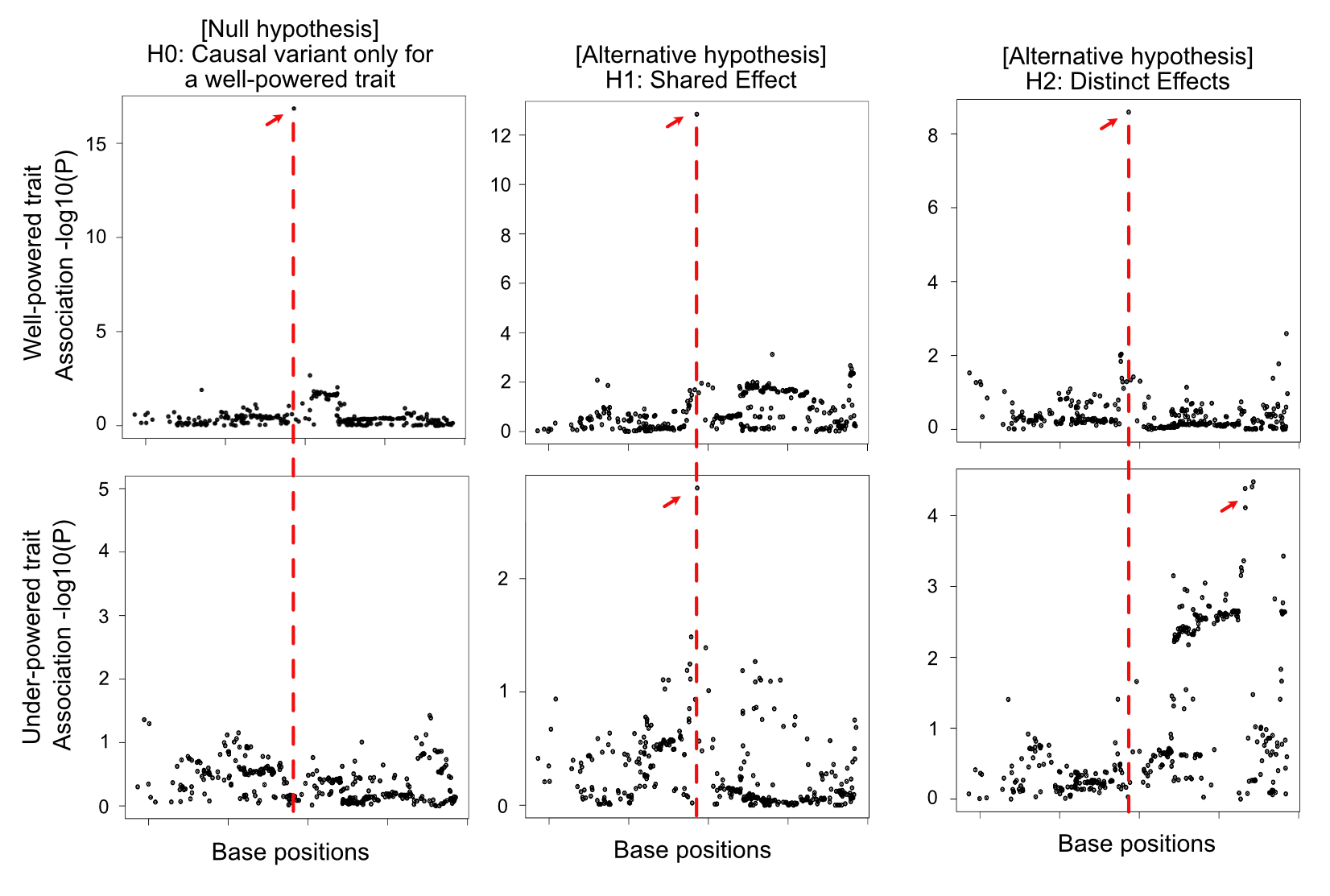

Supplement: S1 Fig — Examples of trait pairs simulated under the null (no causal effect for an underpowered trait, H0), shared effect between well-powered and underpowered traits (H1), and distinct effects between two compared traits (H2). H1 and H2 are competing alternative hypotheses. The simulated true causal variants are indicated by red arrows. (TIF) [file pgen.1010557.s002.tif]

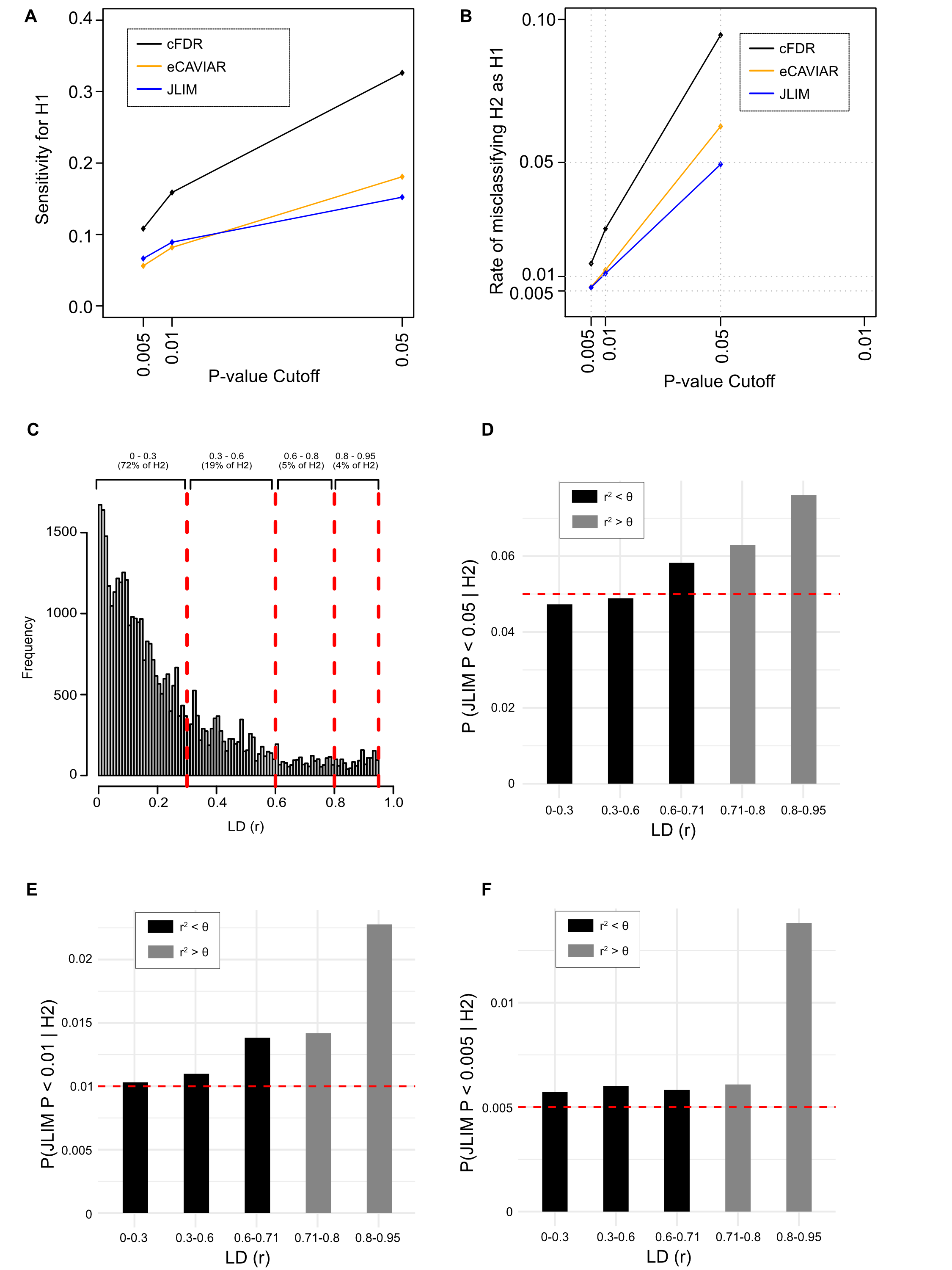

Supplement: S2 Fig — (A) Sensitivity to detect H1 at the same false positive rate for H0. The same p-value cutoff was used for JLIM and cFDR. For eCAVIAR, a posterior cutoff was calibrated to match the p-value cutoff using unfiltered H0 simulation data. (B) The rate of misclassifying H2 as H1. H2 loci include all loci simulating distinct causative variants in LD between 0 to 0.95. Again, the cutoffs of cFDR, eCAVIAR and JLIM were calibrated to the same specificity using H0. (C) The distribution of LD for randomly selected pairs of SNPs. This distribution has been drawn from the LD patterns between random pairs of SNPs within 80 random loci (200kb each) in the population of European ancestry. This distribution was used to simulate the LD between distinct causative variants in H2. (D,E,F) The rate of misclassifying H2 as H1, broken down by the LD between simulated distinct causative variants for two traits. The cutoff of JLIM p-values was set to (D) 0.05, (E) 0.01 or (F) 0.005. The dashed horizontal line indicates the JLIM p-value cutoff. θ represents the genetic resolution parameter for JLIM, set to 0.5 in this study. JLIM does not claim to distinguish H2 beyond the specified genetic resolution limit (r2 > θ; light grey bars). (TIF) [file pgen.1010557.s003.tif]

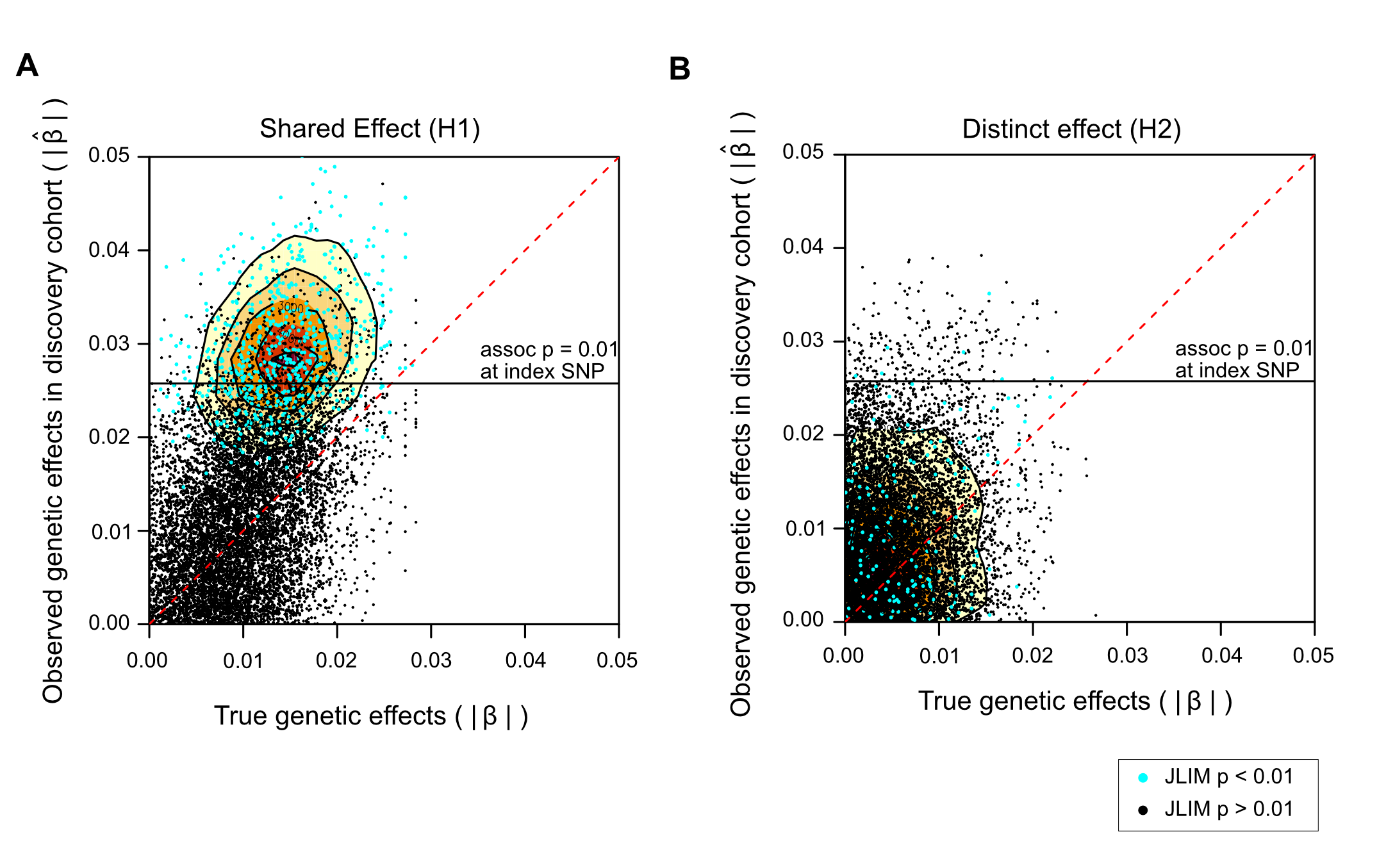

Supplement: S3 Fig — The true and observed genetic effect sizes for underpowered traits are shown to highlight the winner’s curse in a discovery cohort. We show data from 10,000 loci each simulated under (A) H1 and (B) H2. In all panels, each dot represents the genetic effects measured at the index SNPs. The index SNPs are defined as the lead SNPs of association to well-powered clinical traits. Occasionally, the index SNPs deviate from the causative SNPs due to sampling noise, and when this happens, we calculated the true effect size at the index SNP by multiplying the true effect of the causative SNP by the LD between the index and causative SNPs. The loci detected at JLIM p < 0.01 are indicated by cyan dots, and their density distribution are shown in contours. The loci found by cFDR (association p < 5 x 10^−8^ for well-powered trait and < 0.01 for underpowered trait) are represented by dots above the black horizontal line. (TIF) [file pgen.1010557.s004.tif]

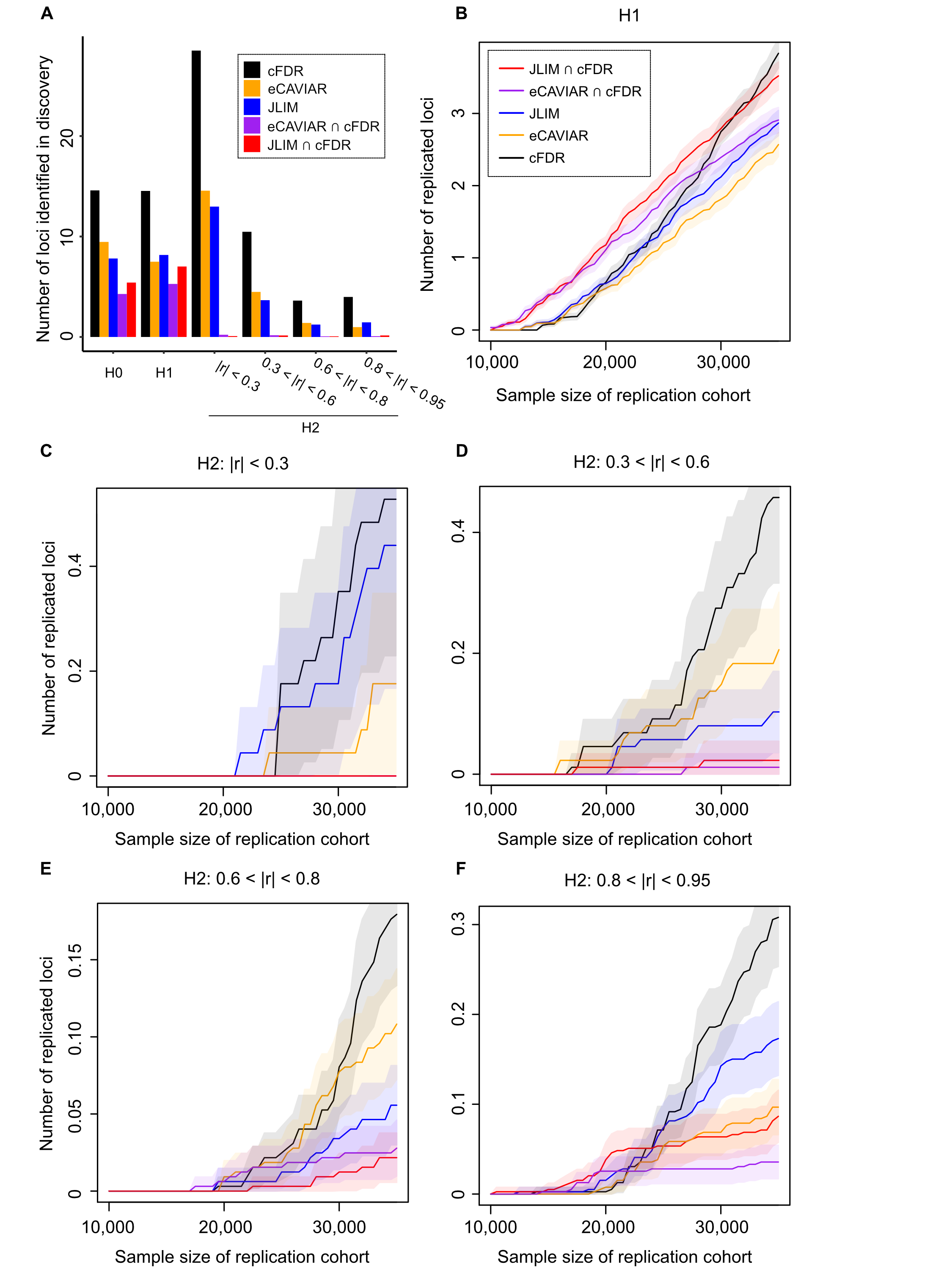

Supplement: S4 Fig — (A) The number of loci identified in a discovery cohort by pleiotropy analysis. (B-F) The number of loci replicated in a validation cohort, subdivided by the configuration of causative variants: (B) H1, H2 with the LD between causative variants to be in the ranges of (C) |r| < 0.3, (D) 0.3 < |r| < 0.6, (E) 0.6 < |r| < 0.8 and (F) 0.8 < |r| < 0.95. In all panels, simulation was conducted under the following parameters: A total of 2,500 association peaks from well-powered GWAS studies (n = 150,000) were tested for pleiotropy in simulated discovery cohorts (n = 10,000), and then the candidate pleiotropic loci were tested for replication in simulated validation cohorts of the same genetic ancestry (n = 10,000–35,000). The candidate loci were identified by conditional false discovery rate (cFDR), eCAVIAR, Joint Likelihood Mapping (JLIM), or the intersection of eCAVIAR or JLIM and cFDR, all at the p-value cutoff of 0.01 (or equivalent posterior cutoff). The 2,500 GWAS peaks consist of the loci simulating no causal effect for underpowered traits (H0) and those simulating the same causal effect between two traits (H1) or distinct causal effects (H2). The proportion of H0 was set to 30%, and the remaining 70% of loci were split to H1 and H2 at the ratio of 1:19. The effect sizes of causative variants are correlated (ρ = 0.7) under H1 but uncorrelated under H2. Bonferroni correction was applied on replication tests. The shaded area denotes the 95% CIs. (TIF) [file pgen.1010557.s005.tif]

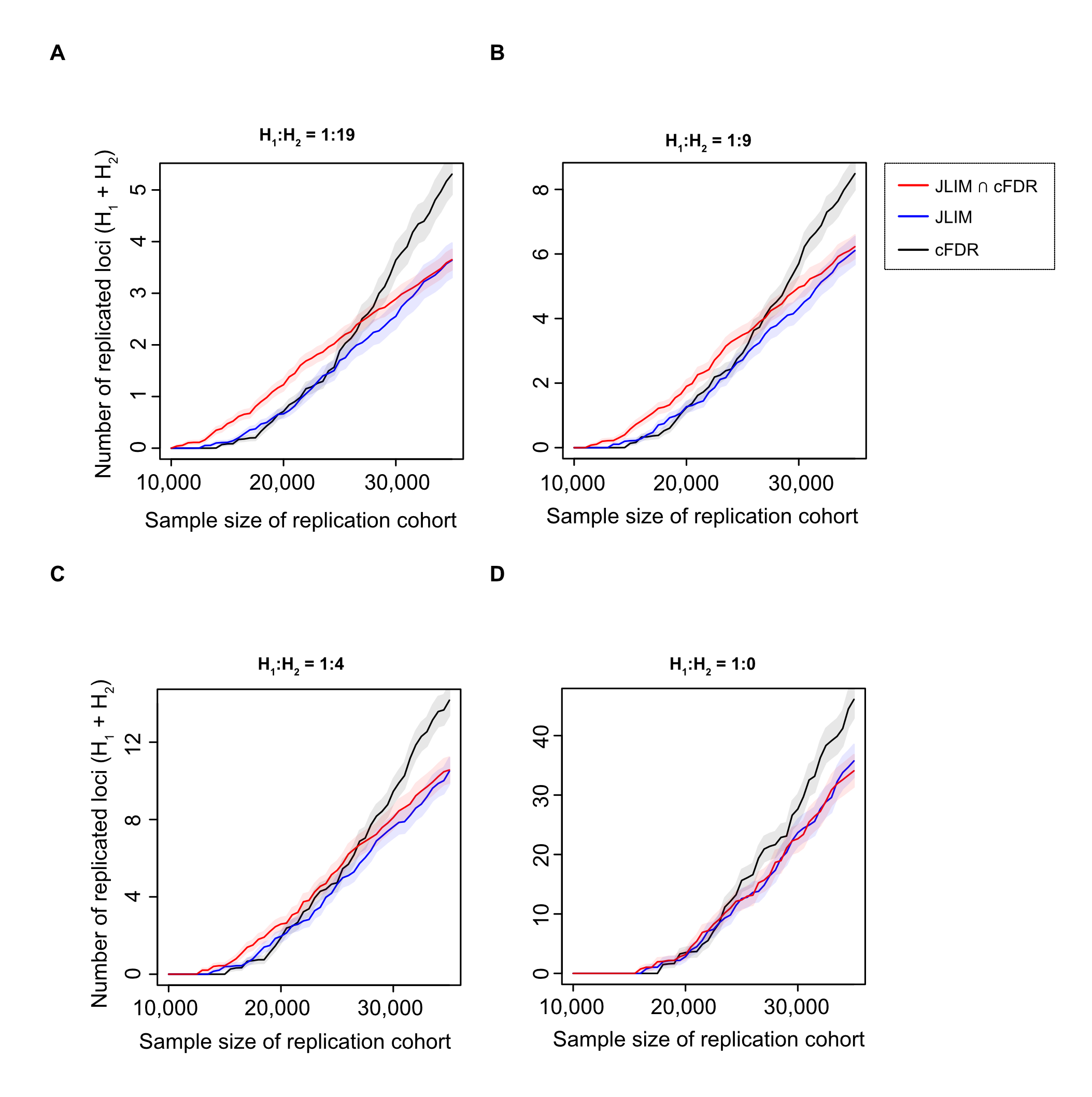

Supplement: S5 Fig — A total of 2,500 association peaks from well-powered GWAS studies (n = 150,000) were tested for pleiotropy in a discovery cohort (n = 10,000), and then the candidate pleiotropic loci were tested for replication in an independent validation cohort of the same genetic ancestry (n = 10,000–35,000). The candidate loci were identified by conditional false discovery rate (cFDR), Joint Likelihood Mapping (JLIM), or the intersection of both, all at the p-value cutoff of 0.01. The 2,500 GWAS peaks consist of the loci simulating no causal effect for underpowered traits (H0) and those simulating the same causal effect between two traits (H1) or distinct causal effects (H2). The proportion of H0 was set to 30%, and the remaining 70% of loci were split to H1 and H2 at the ratio of (A) 1:19, (B) 1:9, (C) 1:4, and (D) 1:0. The effect sizes of causative variants are correlated (ρ = 0.7) under H1 but uncorrelated under H2. Bonferroni correction was applied on replication tests. The shaded area denotes the 95% CIs. (TIF) [file pgen.1010557.s006.tif]

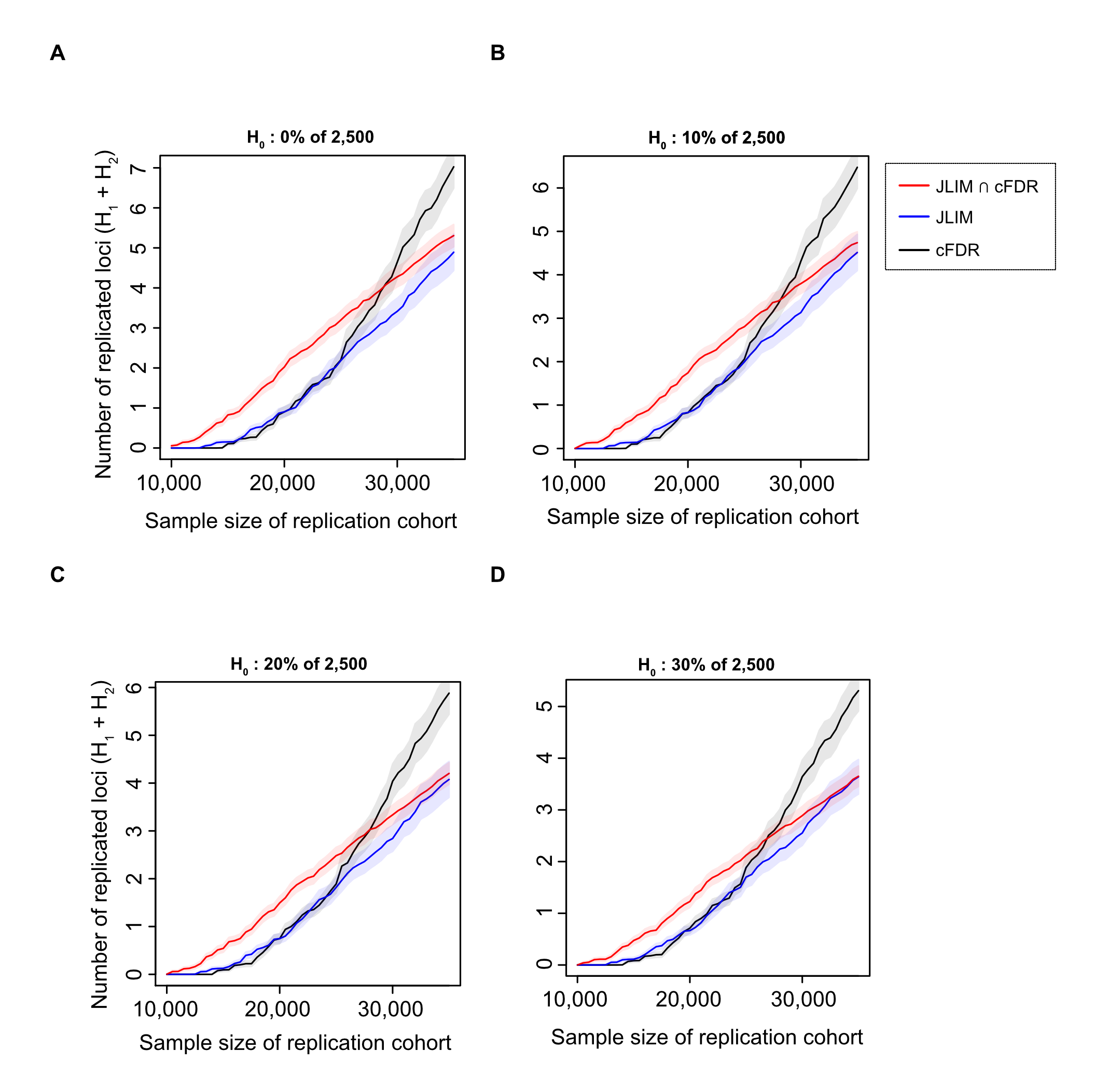

Supplement: S6 Fig — A total of 2,500 association peaks from well-powered GWAS studies (n = 150,000) were tested for pleiotropy in a discovery cohort (n = 10,000), and then the candidate pleiotropic loci were tested for replication in an independent validation cohort of the same genetic ancestry (n = 10,000–35,000). The candidate loci were identified by conditional false discovery rate (cFDR), Joint Likelihood Mapping (JLIM), or the intersection of both, all at the p-value cutoff of 0.01. The 2,500 GWAS peaks consist of the loci simulating no causal effect for underpowered traits (H0) and those simulating the same causal effect between two traits (H1) or distinct causal effects (H2). The proportion of H0 was varied to (A) 0%, (B) 10%, (C) 20% and (D) 30%, and the remaining loci were split to H1 and H2 at the ratio of 1:19. The effect sizes of causative variants are correlated (ρ = 0.7) under H1 but uncorrelated under H2. Bonferroni correction was applied on replication tests. The shaded area denotes the 95% CIs. (TIF) [file pgen.1010557.s007.tif]

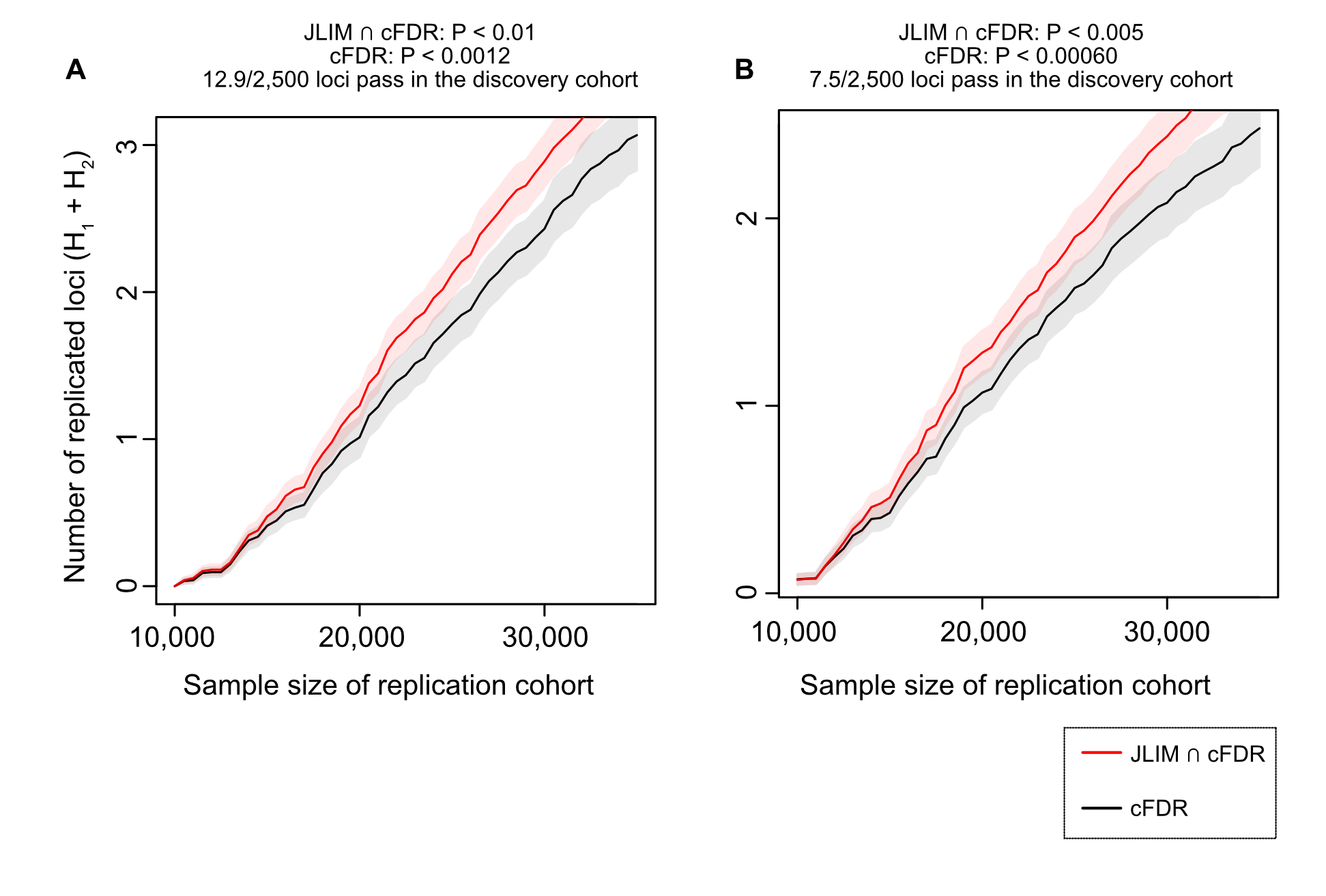

Supplement: S7 Fig — A total of 2,500 association peaks from well-powered GWAS studies (n = 150,000) were tested for pleiotropy in a discovery cohort (n = 10,000), and then the candidate pleiotropic loci were tested for replication in an independent validation cohort of the same genetic ancestry (n = 10,000–35,000). The candidate loci were identified by conditional false discovery rate (cFDR) or by the JLIM/cFDR consensus method. (A) The consensus method (red line) selected 12.9 candidate pleiotropic loci by taking the intersection between JLIM p < 0.01 and cFDR (association p < 0.01 for an underpowered trait). For the comparison, we tightened cFDR threshold to underpowered trait assoc p < 0.0012 so to identify the same number of candidate loci in a discovery cohort (black line). (B) Similarly, the consensus method (red line) found 7.5 candidate loci by taking the intersection between JLIM p < 0.005 and cFDR (assoc p < 0.005 for underpowered trait). The cFDR threshold was tightened to underpowered trait assoc p < 0.00060 for the same number of candidates (black line). In both panels, the 2,500 GWAS peaks consist of the loci simulating no causal effect for underpowered traits (H0) and those simulating the same causal effect between two traits (H1) or distinct causal effects (H2). The proportion of H0 was set to 30%, and the remaining 70% of loci were split to H1 and H2 at the ratio of 1:19. The effect sizes of causative variants are correlated (ρ = 0.7) under H1 but uncorrelated under H2. Bonferroni correction was applied on replication tests. The shaded area denotes the 95% CIs. (TIF) [file pgen.1010557.s008.tif]

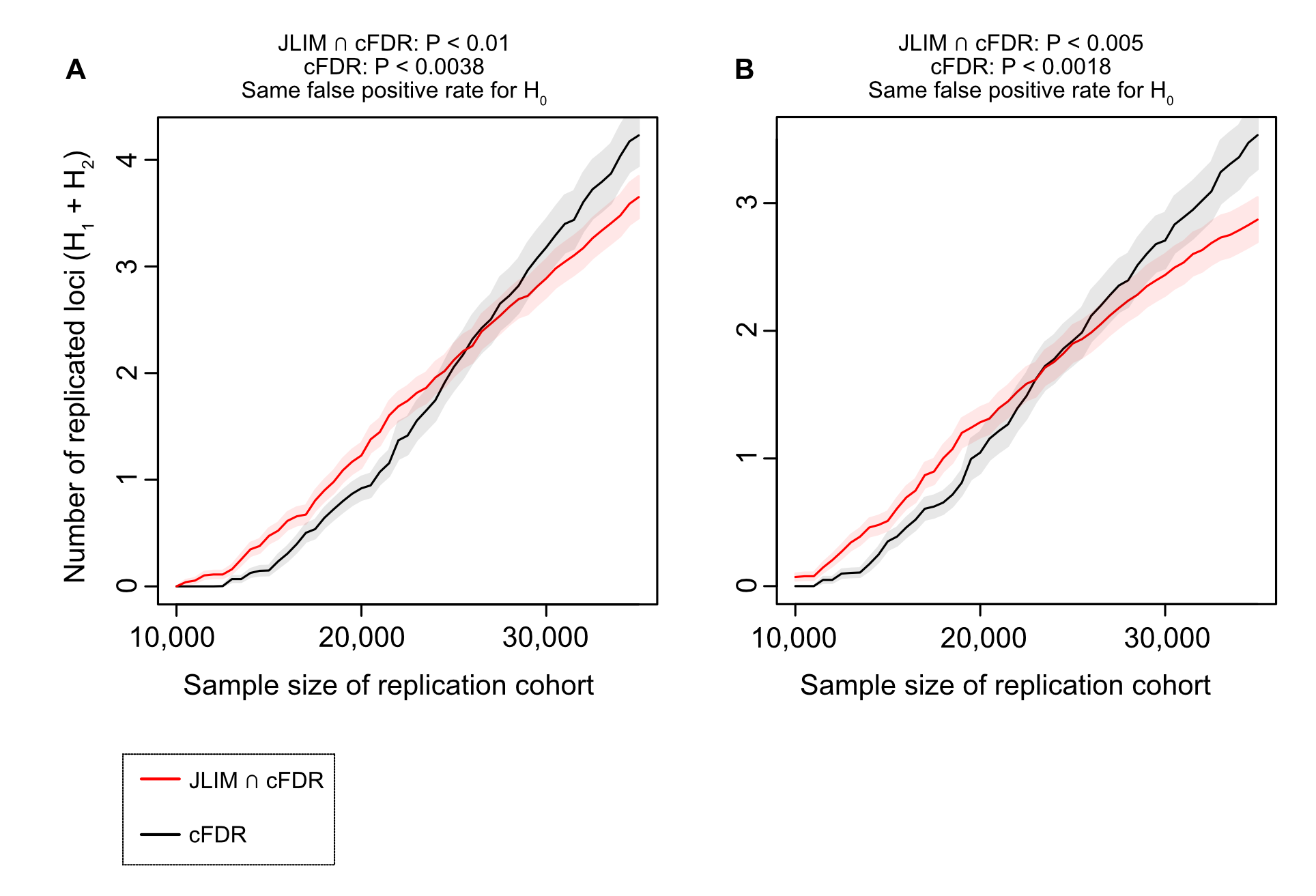

Supplement: S8 Fig — A total of 2,500 association peaks from well-powered GWAS studies (n = 150,000) were tested for pleiotropy in a discovery cohort (n = 10,000), and then the candidate pleiotropic loci were tested for replication in an independent validation cohort of the same genetic ancestry (n = 10,000–35,000). The candidate loci were identified by conditional false discovery rate (cFDR) or by the JLIM/cFDR consensus method. (A) The consensus method (red line), by taking the intersection between JLIM p < 0.01 and cFDR p < 0.01, showed the empirical false positive rate of 0.0038 in simulated H0 dataset. To match this false positive rate, we tightened cFDR threshold to p < 0.0038 (black line). (B) Similarly, the consensus method (red line) showed the empirical false positive rate of 0.0018 in H0 when the intersection was taken at JLIM and cFDR p < 0.005. To match the false positive rate, the cFDR threshold was tightened to p < 0.0018 (black line). The cFDR p-value refers to the p-value of association to an underpowered trait. In both panels, the 2,500 GWAS peaks consist of the loci simulating no causal effect for underpowered traits (H0) and those simulating the same causal effect between two traits (H1) or distinct causal effects (H2). The proportion of H0 was set to 30%, and the remaining 70% of loci were split to H1 and H2 at the ratio of 1:19. The effect sizes of causative variants are correlated (ρ = 0.7) under H1 but uncorrelated under H2. Bonferroni correction was applied on replication tests. The shaded area denotes the 95% CIs. (TIF) [file pgen.1010557.s009.tif]

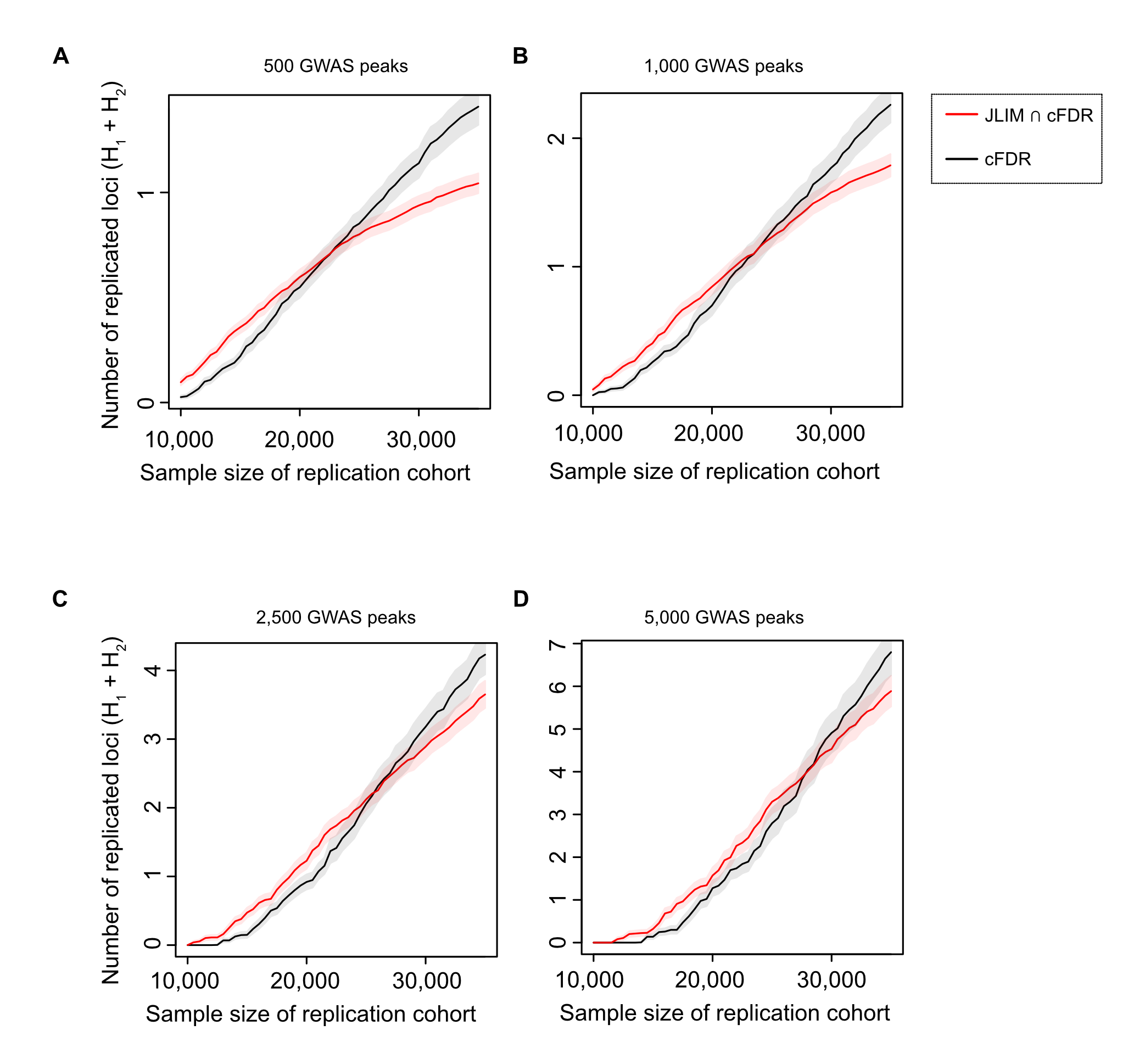

Supplement: S9 Fig — A total of (A) 500, (B) 1,000, (C) 2,500 and (D) 5,000 association peaks from well-powered GWAS studies (n = 150,000) were tested for pleiotropy in a discovery cohort (n = 10,000), and then the candidate pleiotropic loci were tested for replication in an independent validation cohort of the same genetic ancestry (n = 10,000–35,000). The candidate loci were identified by conditional false discovery rate (cFDR) or by the JLIM/cFDR consensus method. The consensus method (red line), by taking the intersection between JLIM p < 0.01 and cFDR p < 0.01, showed the empirical false positive rate of 0.0038 in simulated H0 dataset. To match this false positive rate, we tightened cFDR threshold to p < 0.0038 (black line). The cFDR p-value refers to the p-value of association to an underpowered trait. The GWAS peaks consist of the loci simulating no causal effect for underpowered traits (H0) and those simulating the same causal effect between two traits (H1) or distinct causal effects (H2). The proportion of H0 was set to 30%, and the remaining 70% of loci were split to H1 and H2 at the ratio of 1:19. The effect sizes of causative variants are correlated (ρ = 0.7) under H1 but uncorrelated under H2. Bonferroni correction was applied on replication tests. The shaded area denotes the 95% CIs. (TIF) [file pgen.1010557.s010.tif]

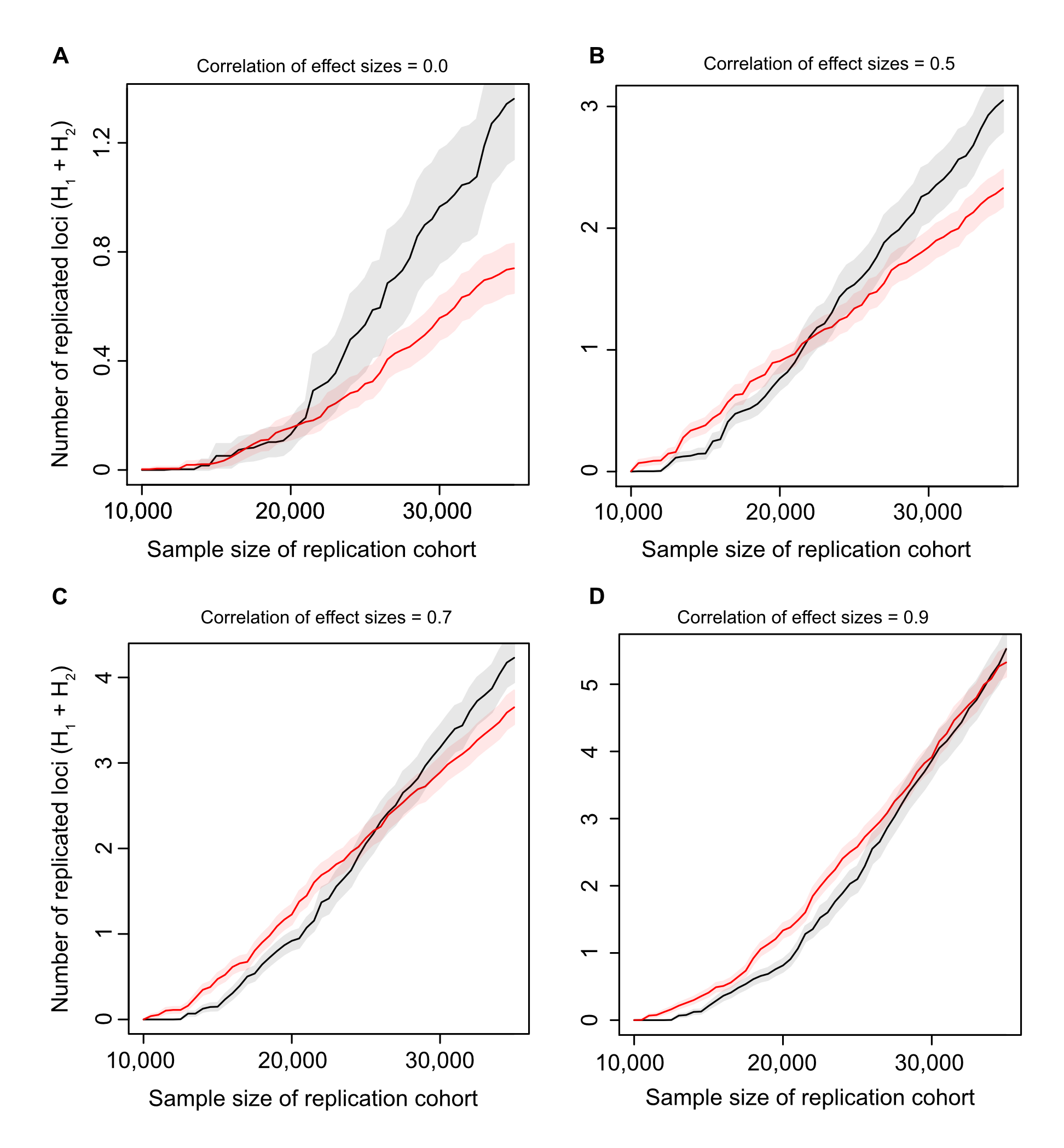

Supplement: S10 Fig — A total of 2,500 association peaks from well-powered GWAS studies (n = 150,000) were tested for pleiotropy in a discovery cohort (n = 10,000), and then the candidate pleiotropic loci were tested for replication in an independent validation cohort of the same genetic ancestry (n = 10,000–35,000). The candidate loci were identified by conditional false discovery rate (cFDR) or by the JLIM/cFDR consensus method. The consensus method (red line), by taking the intersection between JLIM p < 0.01 and cFDR p < 0.01, showed the empirical false positive rate of 0.0038 in simulated H0 dataset. To match this false positive rate, we tightened cFDR threshold to p < 0.0038 (black line). The cFDR p-value refers to the p-value of association to an underpowered trait. The 2,500 GWAS peaks consist of the loci simulating no causal effect for underpowered traits (H0) and those simulating the same causal effect between two traits (H1) or distinct causal effects (H2). The proportion of H0 was set to 30%, and the remaining 70% of loci were split to H1 and H2 at the ratio of 1:19. The effect sizes of causative variants are correlated with (A) ρ = 0.0, (B) ρ = 0.5, (C) ρ = 0.7 and (D) ρ = 0.9 under H1 but uncorrelated under H2. In all panels, Bonferroni correction was applied on replication tests. The shaded area denotes the 95% CIs. (TIF) [file pgen.1010557.s011.tif]

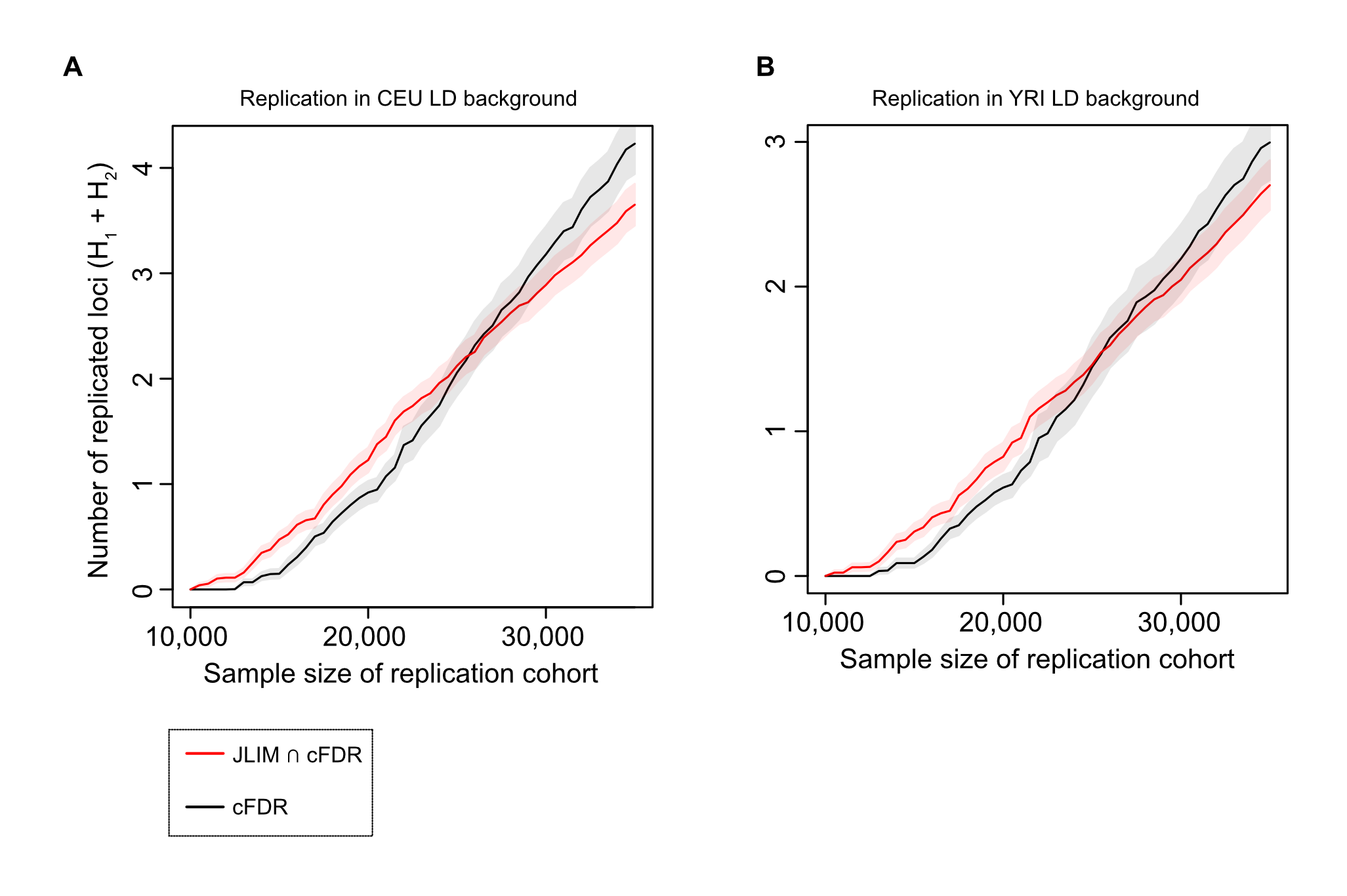

Supplement: S11 Fig — A total of 2,500 association peaks from well-powered GWAS studies (n = 150,000) were tested for pleiotropy in a discovery cohort (n = 10,000), and then the candidate pleiotropic loci were tested for replication in an independent validation cohort of (A) the same (CEU) and (B) different (YRI) genetic ancestry (n = 10,000–35,000). The LD patterns of genetic ancestry were obtained from the 1000 Genomes Project data. In both panels, the candidate loci were identified by conditional false discovery rate (cFDR) or by the JLIM/cFDR consensus method. The consensus method (red line), by taking the intersection between JLIM p < 0.01 and cFDR p < 0.01, showed the empirical false positive rate of 0.0038 in simulated H0 dataset. To match this false positive rate, we tightened cFDR threshold to p < 0.0038 (black line). The cFDR p-value refers to the p-value of association to an underpowered trait. The 2,500 GWAS peaks consist of the loci simulating no causal effect for underpowered traits (H0) and those simulating the same causal effect between two traits (H1) or distinct causal effects (H2). The proportion of H0 was set to 30%, and the remaining 70% of loci were split to H1 and H2 at the ratio of 1:19. The effect sizes of causative variants are correlated with ρ = 0.7 under H1 but uncorrelated under H2. Bonferroni correction was applied on replication tests. The shaded area denotes the 95% CIs. (TIF) [file pgen.1010557.s012.tif]

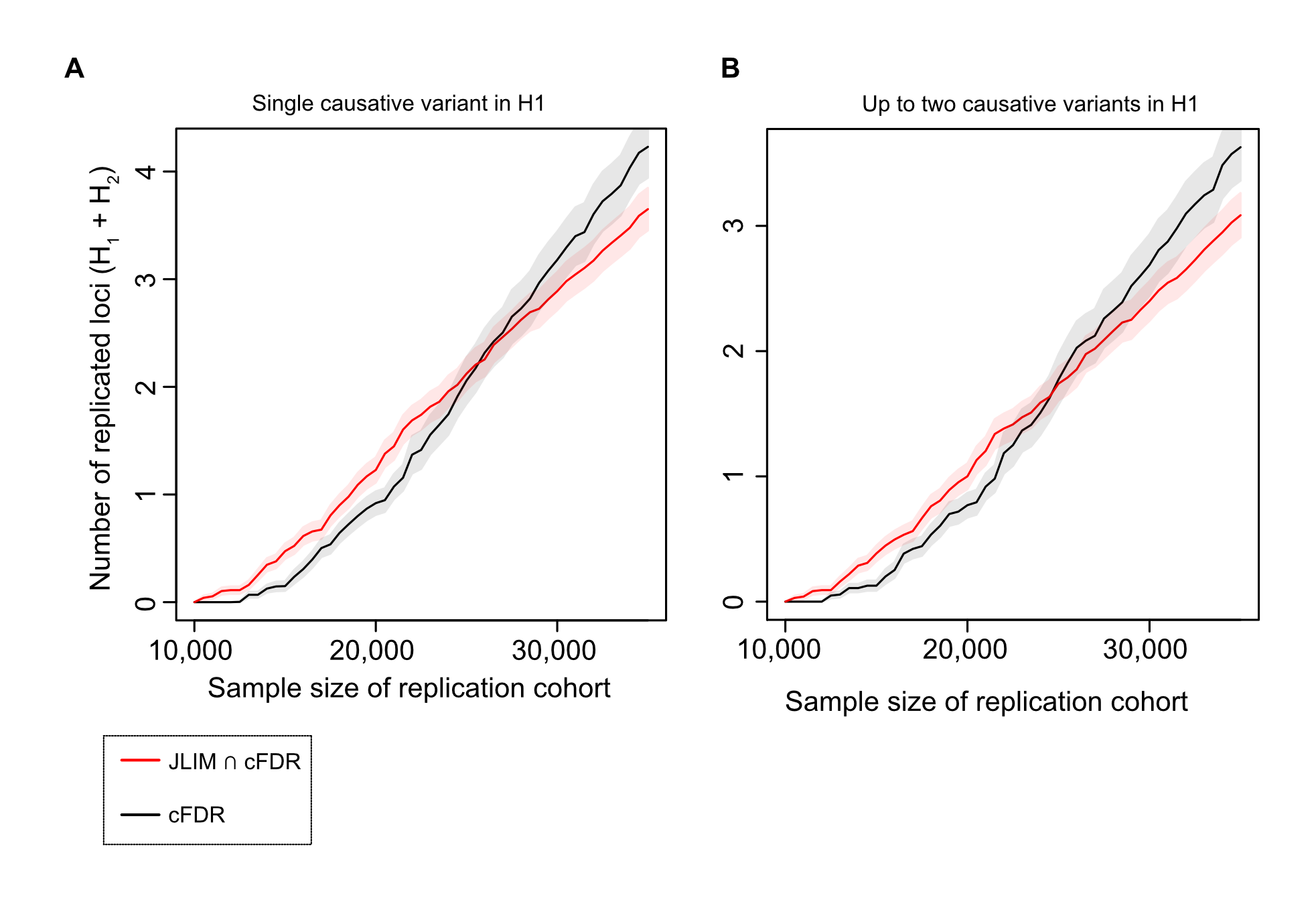

Supplement: S12 Fig — A total of 2,500 association peaks from well-powered GWAS studies (n = 150,000) were tested for pleiotropy in a discovery cohort (n = 10,000), and then the candidate pleiotropic loci were tested for replication in an independent validation cohort of the same genetic ancestry (n = 10,000–35,000). The candidate loci were identified by conditional false discovery rate (cFDR) or by the JLIM/cFDR consensus method. The consensus method (red line), by taking the intersection between JLIM p < 0.01 and cFDR p < 0.01, showed the empirical false positive rate of 0.0038 in simulated H0 dataset. To match this false positive rate, we tightened cFDR threshold to p < 0.0038 (black line). The cFDR p-value refers to the p-value of association to an underpowered trait. The 2,500 GWAS peaks consist of the loci simulating no causal effect for underpowered traits (H0) and those simulating the same causal effect between two traits (H1) or distinct causal effects (H2). The proportion of H0 was set to 30%, and the remaining 70% of loci were split to H1 and H2 at the ratio of 1:19. In Panel (A), only one causative variant was simulated for H1, whereas in Panel (B), up to two causative variants were simulated for H1. The proportion of loci with two causative variants was set to 1/4 of all H1 as expected under Poisson distribution with the causal fraction of 0.01. The effect sizes of causative variants are correlated with ρ = 0.7 under H1 but uncorrelated under H2. Bonferroni correction was applied on replication tests. The shaded area denotes the 95% CIs. (TIF) [file pgen.1010557.s013.tif]

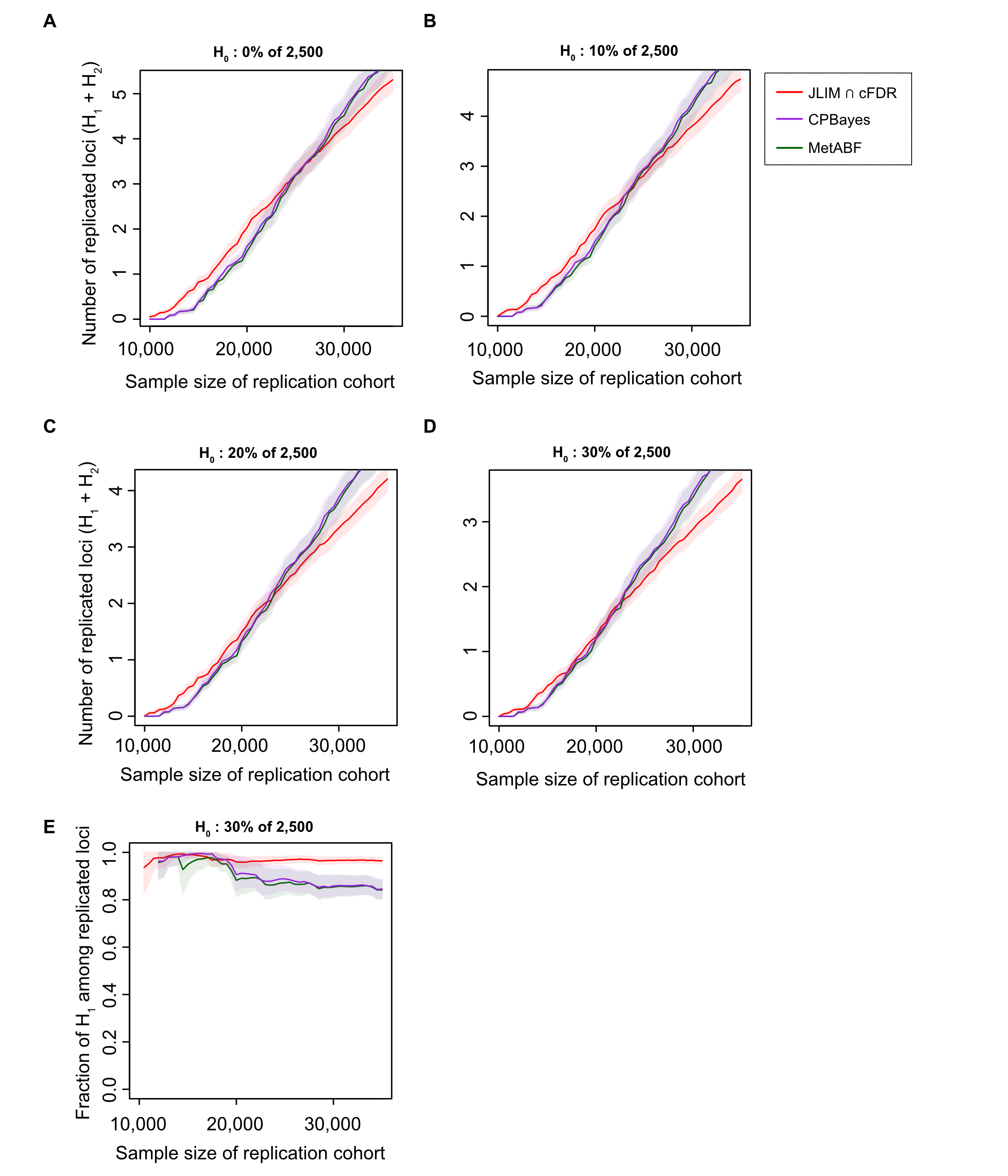

Supplement: S13 Fig — A total of 2,500 association peaks from well-powered GWAS studies (n = 150,000) were tested for pleiotropy in a discovery cohort (n = 10,000), and then the candidate pleiotropic loci were tested for replication in an independent validation cohort of the same genetic ancestry (n = 10,000–35,000). The candidate loci were identified by the JLIM/cFDR consensus method, MetABF and CPBayes. The consensus method (red line), by taking the intersection between JLIM p < 0.01 and cFDR p < 0.01, showed the empirical false positive rate of 0.0038 in simulated H0 dataset. Bayesian posterior thresholds for MetABF (green) and CPBayes (purple) calibrated using H0 loci to match the false positive rate of the consensus method. The 2,500 GWAS peaks consist of the loci simulating no causal effect for underpowered traits (H0) and those simulating the same causal effect between two traits (H1) or distinct causal effects (H2). The proportion of H0 varied to (A) 0%, (B) 10%, (C) 20% and (D) 30%, and the remaining loci were split to H1 and H2 at the ratio of 1:19. In all panels, the effect sizes of causative variants are correlated with ρ = 0.7 under H1 but uncorrelated under H2. Bonferroni correction was applied on replication tests. The shaded area denotes the 95% CIs. In (E), the fraction of H1 among replicated loci is compared among three methods when the proportion of H0 is 30% (similar for different proportions of H0). (TIF) [file pgen.1010557.s014.tif]

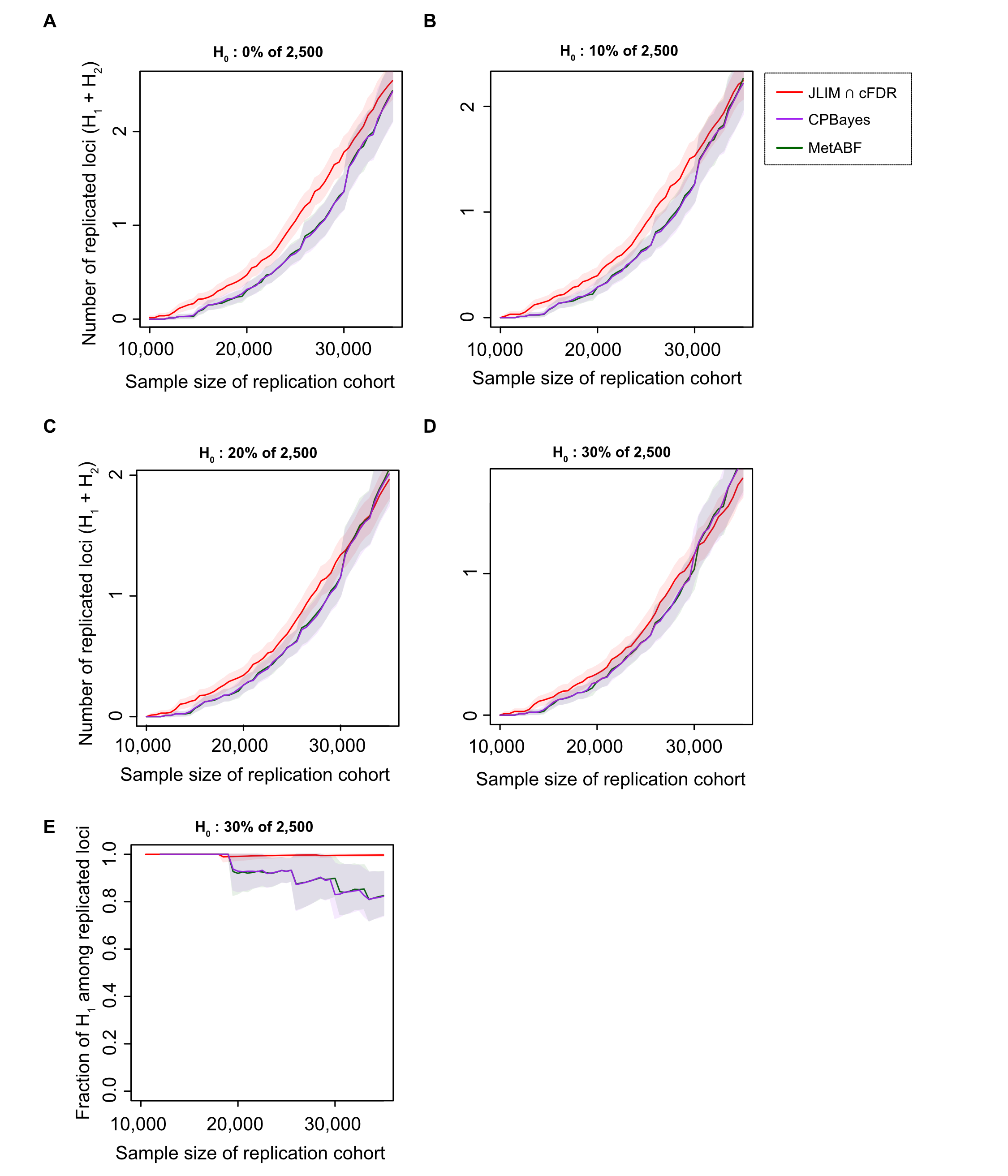

Supplement: S14 Fig — A total of 2,500 association peaks from well-powered GWAS studies (n = 150,000) were tested for pleiotropy in a discovery cohort (n = 10,000), and then the candidate pleiotropic loci were tested for replication in an independent validation cohort of the different genetic ancestry (YRI; n = 10,000–35,000). The candidate loci were identified by the JLIM/cFDR consensus method, MetABF and CPBayes. The consensus method (red line), by taking the intersection between JLIM p < 0.01 and cFDR p < 0.01, showed the empirical false positive rate of 0.0038 in simulated H0 dataset. Bayesian posterior thresholds for MetABF (green) and CPBayes (purple) calibrated using H0 loci to match the false positive rate of the consensus method. The 2,500 GWAS peaks consist of the loci simulating no causal effect for underpowered traits (H0) and those simulating the same causal effect between two traits (H1) or distinct causal effects (H2). The proportion of H0 varied to (A) 0%, (B) 10%, (C) 20% and (D) 30%, and the remaining loci were split to H1 and H2 at the ratio of 1:19. In all panels, the effect sizes of causative variants are correlated with ρ = 0.7 under H1 but uncorrelated under H2. Bonferroni correction was applied on replication tests. The shaded area denotes the 95% CIs. In (E), the fraction of H1 among replicated loci is compared among three methods when the proportion of H0 is 30% (similar for different proportions of H0). (TIF) [file pgen.1010557.s015.tif]

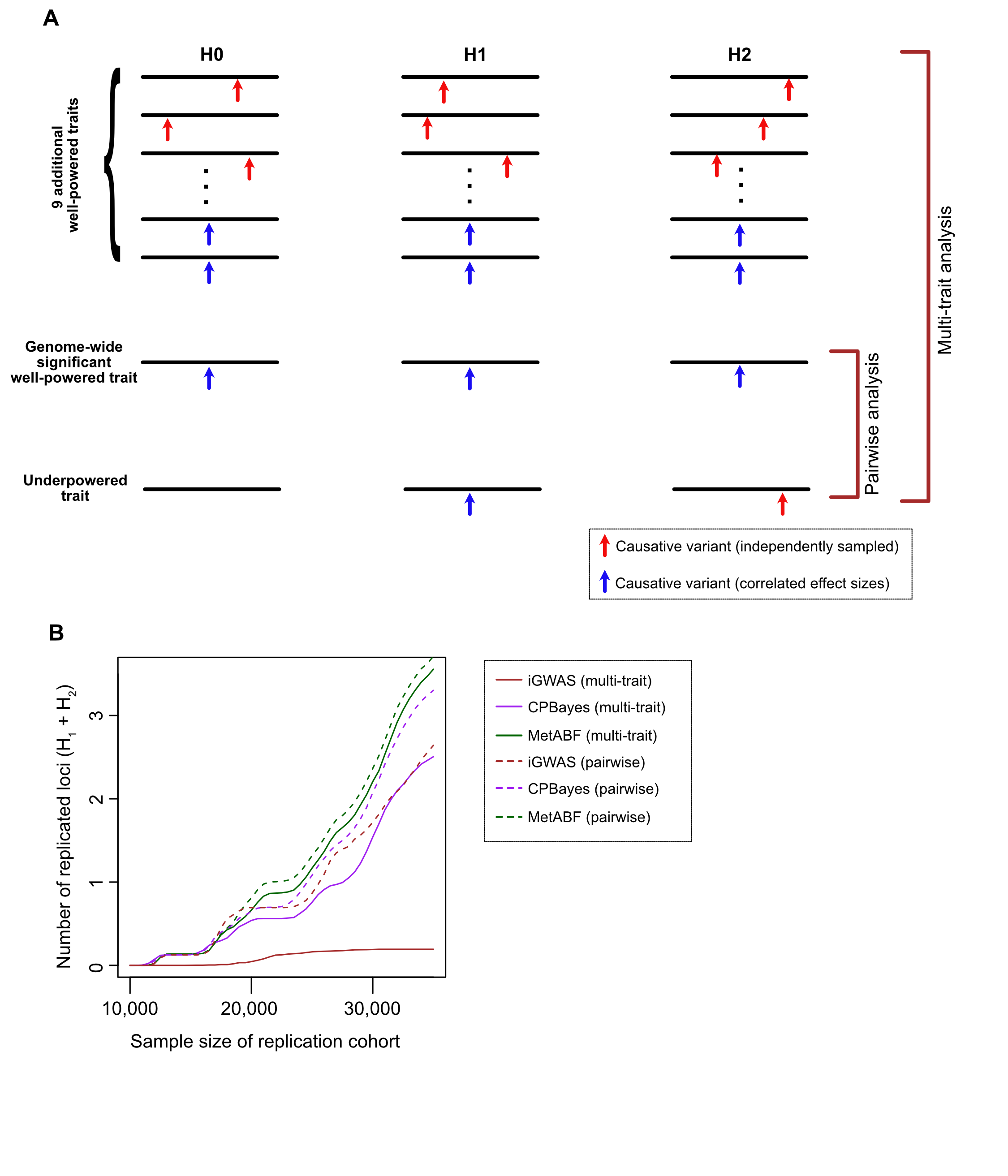

Supplement: S15 Fig — Ten well-powered traits and one underpowered trait were simulated under H0, H1 and H2. One of the well-powered traits is ascertained to have a genome-wide significant association peak, and the rest of well-powered traits were randomly decided to have the same or distinct causative variants by sampling from a binomial distribution Binom(n = 9, p = 1/20). The underpowered trait harbors no causative variant, the same causative variant or distinct causative variant depending on whether it is simulating H0, H1 or H2. Effect sizes of all traits sharing the same causative variant were sampled together from a multivariate normal distribution with the correlation parameter of 0.7. Effect sizes of traits simulating distinct causative variants were sampled independently. GWAS association statistics at the focal SNP were generated with the sample sizes of n = 150,000 for the well-powered traits and n = 10,000 for the underpowered trait. The validation cohort of the same ancestry was simulated with the sample sizes of n = 10,000 to 35,000. From these simulated H0, H1 and H2 loci, a total of 2,500 GWAS loci were randomly selected at the proportions of 30%, 3.5% and 66.5%, respectively (H1:H2 ratio of 1:19). (B) We applied iGWAS, CPBayes and MetABF on these data. Dashed lines indicate pairwise analyses for which only one genome-wide significant well-powered trait was compared with an underpowered trait. Solid lines indicate multi-trait analyses using the full set of ten well-powered traits and one underpowered trait. Candidate pleiotropic loci were selected for replication analysis at the cutoffs of p-value of 0.01 (iGWAS), or equivalent posterior probability thresholds calibrated in the H0 dataset (CPBayes and MetABF). Bonferroni correction was applied on replication tests in the validation cohort. (TIF) [file pgen.1010557.s016.tif]

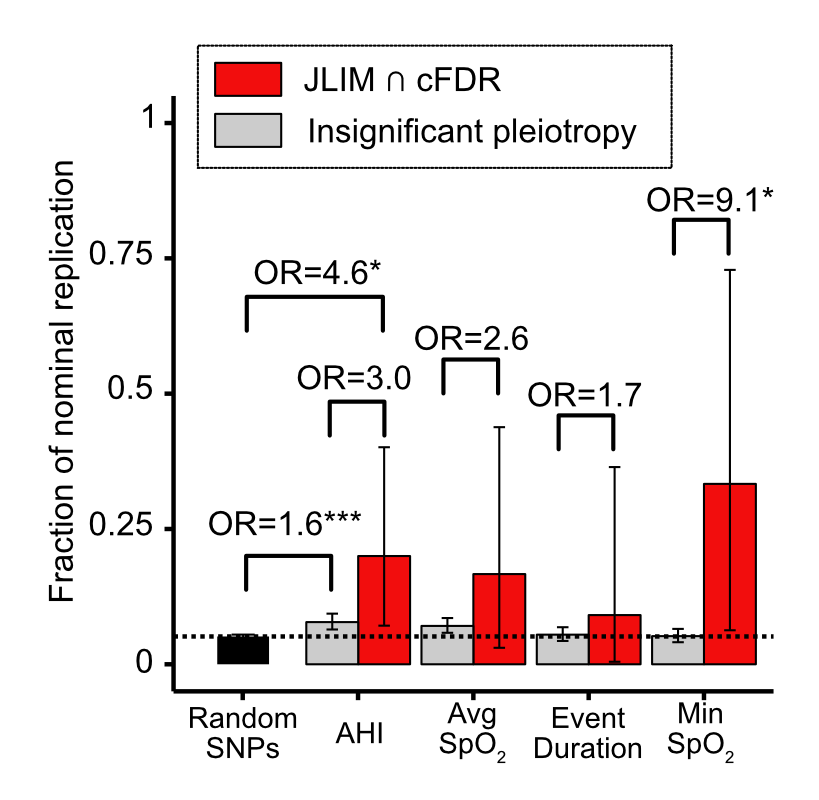

Supplement: S16 Fig — The plot shows the fractions of randomly selected and putative pleiotropic loci with OSA associations that are nominally replicated in the meta-analyzed independent validation cohort (S6 Table). (TIF) [file pgen.1010557.s017.tif]
